# Understanding fragility: implications for global health research and practice

**DOI:** 10.1093/heapol/czz142

**Published:** 2019-12-10

**Authors:** Karin Diaconu, Jennifer Falconer, Nicole Vidal, Fiona O’May, Esther Azasi, Kelly Elimian, Ibrahim Bou-Orm, Cristina Sarb, Sophie Witter, Alastair Ager

**Affiliations:** NIHR Research Unit on Health in Situations of Fragility, Institute for Global Health and Development, Queen Margaret University, Edinburgh, EH21 6UU, UK

**Keywords:** Fragility, resilience, health systems, accountability

## Abstract

Advances in population health outcomes risk being slowed—and potentially reversed—by a range of threats increasingly presented as ‘fragility’. Widely used and critiqued within the development arena, the concept is increasingly used in the field of global health, where its relationship to population health, health service delivery, access and utilization is poorly specified. We present the first scoping review seeking to clarify the meaning, definitions and applications of the term in the global health literature. Adopting the theoretical framework of concept analysis, 10 bibliographic and grey literature sources, and five key journals, were searched to retrieve documents relating to fragility and health. Reviewers screened titles and abstracts and retained documents applying the term fragility in relation to health systems, services, health outcomes and population or community health. Data were extracted according to the protocol; all documents underwent bibliometric analysis. Narrative synthesis was then used to identify defining attributes of the concept in the field of global health. A total of 377 documents met inclusion criteria. There has been an exponential increase in applications of the concept in published literature over the last 10 years. Formal definitions of the term continue to be focused on the characteristics of ‘fragile and conflict-affected states’. However, synthesis indicates diverse use of the concept with respect to: level of application (e.g. from state to local community); emphasis on particular antecedent stressors (including factors beyond conflict and weak governance); and focus on health system or community resources (with an increasing tendency to focus on the interface between two). Amongst several themes identified, trust is noted as a key locus of fragility at this interface, with critical implications for health seeking, service utilization and health system and community resilience.

**Key Messages**

- Previous well-defined applications of fragility relate to ‘fragile and conflict-affected states’.
- Fragility is increasingly used across a broader range of contexts, including politically stable, secure and economically prosperous settings to denote key barriers in achieving health advances.
- Critical to global health and intervention framing, fragility increasingly refers to breakdowns at the interface between the community and the health system.

## Introduction

Globally, there has been a substantial improvement in health outcomes over the last 50 years. For instance, mortality in under-5-year olds has decreased steadily from ∼216 deaths per 1000 live births in 1960 to 38.9 deaths in 2017 (GBD 2017 Mortality Collaborators, 2018). However, estimates for adult age groups, specifically both men and women aged 20–45 years, indicate cause for concern: decreases in mortality rates have largely plateaued and people spend longer lives in poor health, often struggling with socially driven health conditions such as substance use (GBD 2017 Causes of Death Collaborators, 2018). In addition, global mortality due to violence-related causes has increased and notably, while communicable disease-related mortality and morbidity are decreasing or plateauing; the total deaths due to non-communicable diseases and their risk factors have increased by 22.7% between 2007 and 2017 (The Lancet, 2018).

Increasingly, there is wide-ranging recognition that there are major threats to progress towards health and universal health coverage to which ‘neither wealth nor development renders countries immune’ (OECD, 2016, p. 20). Estimates from the Global Burden of Disease Study 2017 illustrate vast regional- and country-level differences in health indicators and suggest that no country is on track to meet the sustainable development goals by 2030 (The Lancet, 2018). Violence and prolonged conflict, political and economic instability, marginalization and inequality, weak and distorted national governance structures and processes (Foreign and Commonwealth Office—UK Government, 2011; OECD, 2016) and substantive environmental threats [including climate change (Watts *et al.*, 2017) and natural disasters (Ijaz *et al.*, 2012; Watts *et al.*, 2017)] are beginning to undermine, and even reverse, the advances in health and well-being achieved within the last half-century (GBD 2017 Causes of Death Collaborators, 2018).

The above threats are increasingly linked to the concept of fragility. The World Bank’s work in characterizing fragility and determining which countries are experiencing particularly fragile situations has been in effect since 2006 (World Bank, 2016), focusing largely on countries experiencing the effects of conflict and violence. The list has undergone several changes since 2006, drawing on increased knowledge on the effects of conflict and violence on development challenges. The World Bank’s ‘fragile situations’ list determines which countries score below a specific cut-off on the Country Policy and Institutional Assessment—a 16-item measure focused on determining a country’s performance across economic management, structural policy, policies for social inclusion and equity and public sector management and institutions (The World Bank Group, 2017). The measure does not focus on health specifically, but it is recognized that the governance and economic-related challenges identified present severe threats also to public and personal health. Currently, 30 countries are assessed as experiencing ‘fragile situations’ based on the Country Policy and Institutional Assessment, with a further four (West Bank and Gaza, Iraq, Lebanon and Libya) identified as ‘fragile’ but not being formally assessed (World Bank, 2019).

In contrast to the World Bank, the OECD views fragility as a more complex and multidimensional phenomenon. In their seminal report series on the topic ‘States of Fragility’, they note that fragility is ‘the combination of exposure to risk and insufficient capacity of the state, system and/or communities to manage, absorb or mitigate those risks. Fragility can lead to negative outcomes including violence, the breakdowns of institutions, displacement, humanitarian crises or other emergencies’ (OECD, 2016). While still conceptualizing fragility as a state- or country-level phenomenon, the OECD expands on the World Bank Country Policy and Institutional Assessment by focusing on forms of fragility, which affect economic, environmental, political, security and societal domains. OECD estimates indicate that by 2030, 80% of the global poor will live in contexts affected by one or more of these drivers of fragility (OECD, 2018); many stable and prosperous environments that fall into the middle-income country bracket are included in this estimate (OECD, 2016). By promoting this more granular understanding of fragility across a wider pool of countries, the OECD hopes to prompt reflection on the differentiated approaches needed to strengthen the coping capacities of diversely fragile contexts.

While these two approaches have clear influence on how the concept of fragility is used, there are indications of its increasingly wide and diverse use as an explanatory factor in global discussions unrelated to state circumstance (e.g. in the UK, in relation to patient engagement and empowerment as described in Martin and Finn, 2011, or in Norway, in relation to the increasingly fragile life of chronic disease patients as described in Jerpseth, 2017). If fragility is to coherently inform the analysis of global health challenges and shape interventions to address them, it is important to work towards a common understanding of the meaning and appropriate application of the concept. This scoping review seeks to comprehensively map the current use of the term in the field of global health and, noting the variety of uses and emphases, establish the core attributes of fragility and their implications for intervention.

## Methods

### Theoretical framework

As per the method of Walker and Avant (1994, p. 38), we interrogate the meaning of a concept via a systematic process, which allows us ‘to distinguish between the defining attributes of a concept and its irrelevant attributes’ (Nuopponen, 2010). Nuopponen (2010) noted that Walker and Avant (1994) distinguish steps necessary for undertaking a comprehensive concept analysis. In this study, we focus on the first four of these steps: selecting a concept to study, determining the aims of the analysis, identifying uses of the term and determining defining attributes of the concept post-analysis of all identified uses. As a scoping review that significantly references empirical work, we integrate references to empirical cases throughout the work rather than through the separate steps specified by Walker and Avant (1994).

### Research aims and process

This review aims to analyse the use of the concept of fragility in the global health literature: identifying where and how the concept has been used, and its existing definitions and applications, to establish a coherent understanding of its potential relevance to global health interventions. To identify all relevant uses of fragility in the global health literature, we conduct a scoping review, extract data according to a pre-specified protocol and, via narrative synthesis, identify attributes describing how and where the concept has been applied and the meanings that have been attached to it.

### Scoping review

The scoping review was undertaken in accordance with the guidance outlined in Arksey and O’Malley (2005) and Colquhoun *et al.* (2014). Before study commencement, search strategies, inclusion and exclusion criteria for study selection, data extraction forms and data analysis plans were developed. All strategies and materials were piloted; inclusion and exclusion criteria and data extraction forms were iteratively refined to capture the full breadth of relevant studies and information (Levac *et al.*, 2010).

### Information sources and searches

Literature searches were conducted across 10 bibliographic databases and grey literature sources between September and October 2017. Bibliographic databases included Medline, CINAHL and Global Index Medicus (formerly Global Health Library). Grey literature sources included repositories and listings held by Health Systems Global, OpenGrey, Grey Literature Report, Management Sciences for Health, Department for International Development, the World Bank and World Health Organization IRIS. The following five key journals were also searched: ‘Conflict and Health, Health Policy and Planning, Health Research Policy and Systems, Global Health: Science and Practice and Social Science and Medicine’. The journals were chosen purposively to capture health systems and global health-relevant literature. Searches were intentionally broad to capture the full range of literature relating to fragility and health. Search strategies were adapted according to the data source and are detailed in Supplementary File 1.

### Study selection

As the first scoping review on this topic, our focus was deliberately broad; no restrictions relating to settings, publication date, types of publications or materials (e.g. presentations, documents, videos) applied. Two reviewers screened titles for relevance and retained those potentially referring to situations, settings or vulnerable populations classed as fragile. The same reviewers then screened abstracts in accordance with the criteria presented in Table 1.

**Table 1:** Inclusion and exclusion criteria

Inclusion criteria	Exclusion criteria
Study includes a statement suggestive of ‘fragility’ or the term ’fragile’ (and synonyms or derivatives)	‘Fragility’ or synonym of ‘fragility’ not used
Above statement used in relation to: health systems and their building blocks (World Health Organization, 2010), services, population health outcomes, or community/population health (or health capacities)—e.g. as in reference to vulnerable populations, factors compromising population health, health status or social protection or financing mechanisms	‘Fragility’ or synonym not used in relation to: health system, services, population health outcomes, or community/population health or health capacities
	Study focused on medical uses of fragility (e.g. bones, genes, technologies)
	Documents where abstract/full text is not available electronically
	Documents not in English

Comprehensive double screening of titles and abstracts was not possible due to time and resource constraints. To ensure consistency in the screening process, the third reviewer independently screened a random selection of 10% of titles and abstracts from each of the sources searched and confirmed the reliable operationalization of selection criteria within the screening of the remaining 90% of identified documents. Disagreements were resolved by discussion.

### Data extraction

Data extraction was undertaken using a standardized and piloted template (Supplementary File 2). Data were extracted in relation to study identifiers (e.g. study author, year of publication), settings (i.e. country, areas or regions under study), methods (e.g. quantitative, qualitative, literature review), findings (i.e. main results as presented in the study) and limitations (both author and reviewer specified). Authors’ definitions of fragility, and any descriptions linked to the concept, were extracted verbatim when available.

### Analysis

As per Walker and Avant (Nuopponen, 2010), we first describe where and how the concept of fragility has been applied. To provide an account of the former, we characterize the reviewed body of literature via bibliometric analyses. To address the latter, we conduct a narrative synthesis (Popay *et al.*, 2006) of extracted data, determining the way fragility has been defined and applied and identifying emerging patterns and ‘defining attributes’ of the concept.

### Reporting

As scoping review reporting guidelines are currently under development, PRISMA reporting standards were followed where possible (Moher *et al.*, 2009); items 12–13, 15–16, 19–20 and 22–23 were not applicable.

## Findings

**Figure 1:**
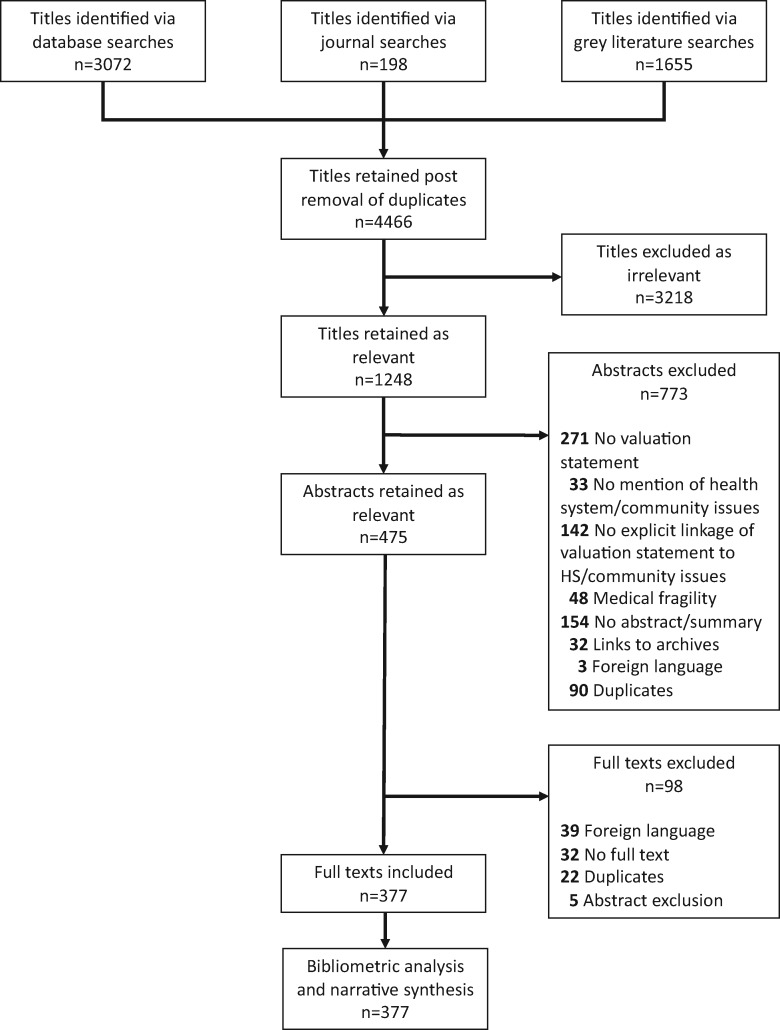
PRISMA diagram showing document selection criteria and methodologies utilized.

Database and grey literature searches retrieved 4466 documents post-deduplication. Figure 1 provides a PRISMA illustration of the study inclusion/exclusion process and analysis approaches used. A total of 377 studies were retained for inclusion in the review. Throughout the text, we refer to scoping review references as SR (see references included in scoping review in Supplementary File 3).

### Bibliometric analysis: identifying contexts where fragility has been used as an explanatory concept in the global health literature

**Figure 2:**
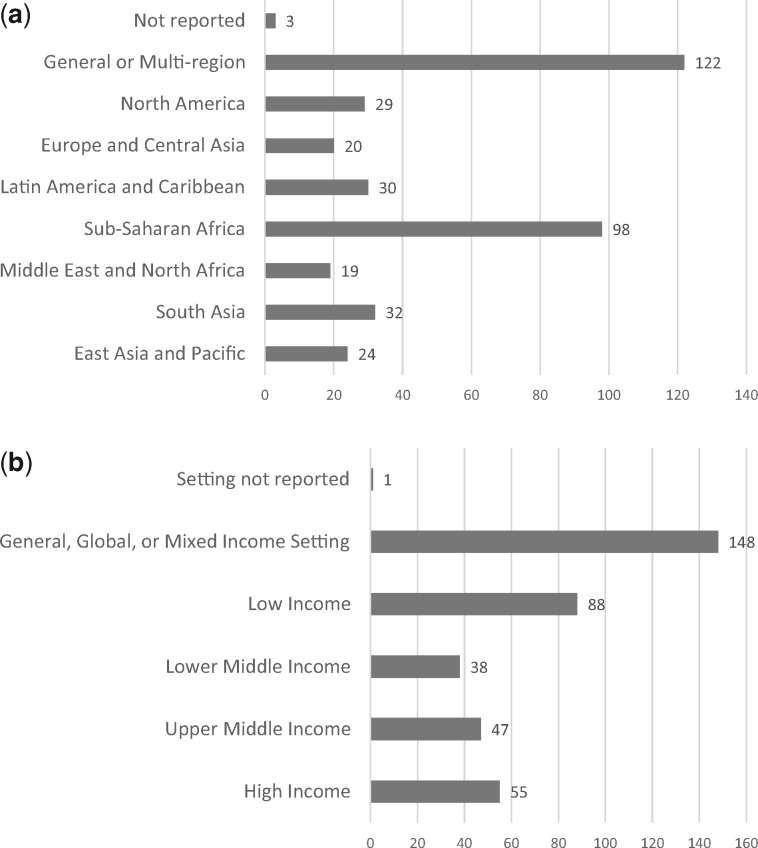
(a) Graphics showing number of included studies according to region (*n* = 377). (b) Graphics showing number of included studies according to income level (*n* = 377).

The 377 studies reported on research conducted across all seven World Bank regions (World Bank, 2018) (Figure 2a) and income levels (Figure 2b). Included studies addressed countries across all income levels: 148 (39%) studies refer to global or mixed-income countries, 88 (23%) studies refer to low-income countries, 38 (10%) studies refer to lower-middle-income countries, 47 (12%) studies refer to upper-middle-income countries and 55 (14%) studies refer to high-income countries.

**Figure 3:**
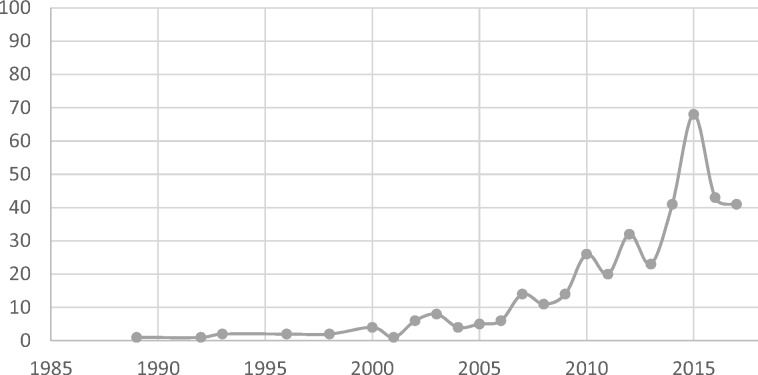
Frequency of included studies by publication year (*n* = 375). Search end date: October 2017.

The earliest study included was published in 1989, and the most recent study was published in 2017 (literature searches were conducted in the early months of 2018). Studies referring to fragility significantly increased in number from the early 2000s, culminating in a peak (*n* = 68) in 2015 (Figure 3). The upswing in publications is across the majority of regions, but the 2015 peak appears largely driven by Sub-Saharan African studies (24 studies published in 2015) and global or multi-region studies (21 studies published in 2015; data not shown).

Included studies spanned a variety of study types and methods (Table 2). A total of 176 (47%) studies were literature, scoping or systematic reviews or commentary and analytic pieces widely drawing on document review. We note the relatively high number of primary studies conducted (45%, *n* = 170), dominated by qualitative studies.

**Table 2:** Number of studies reviewed by method

Primary study: quantitative	46
Primary study: qualitative	94
Primary study: mixed methods	30
Secondary analysis study: quantitative	21
Secondary analysis study: qualitative	7
Secondary analysis study: mixed methods	3
Others: systematic/scoping review	18
Others: literature review/discussion paper	98
Others: report/commentary/letter/news/blog post	60
Total	377

**Figure 4:**
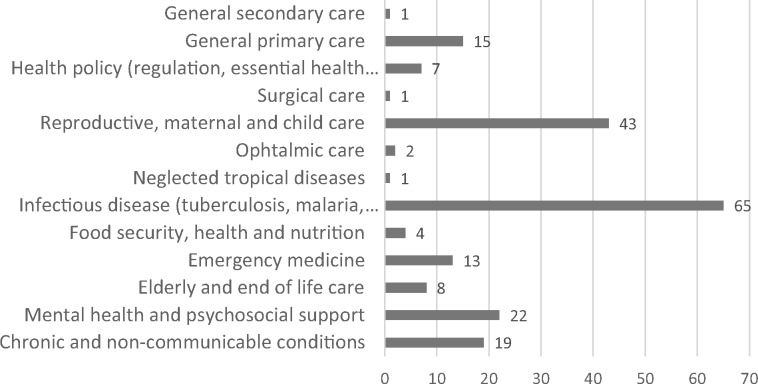
Frequency of included studies by clinical area of focus (*n* = 201).

Of the included studies, 201 (53%) studies focused on a specific clinical area (see Figure 4). Of these studies, 65 (32%) studies related to infectious diseases such as human immunodeficiency virus (HIV)/acquired immune deficiency syndrome, tuberculosis and malaria or disease outbreaks.

**Figure 5:**
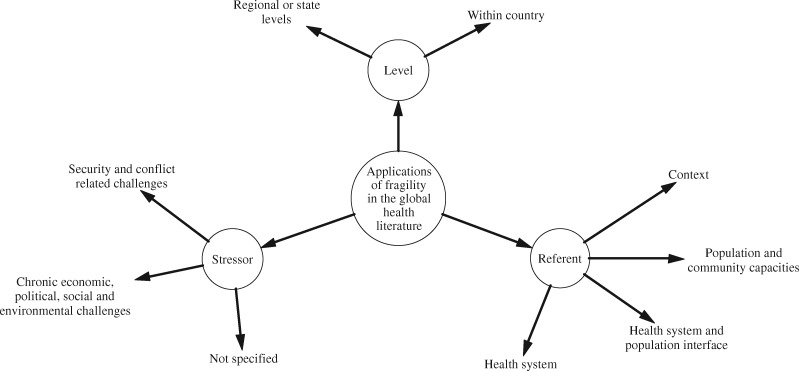
Characterization of how fragility is applied in the global health literature.

Reviewing the use of the concept across this literature distinguished three key dimensions determining its application (see Figure 5). First, fragility is located at differing levels: 204 (54.1%) documents referred to fragility in relation to state (or regional) circumstances, whereas 173 (45.9%) documents referred to within-country phenomena not specifically related to the state or its functions. Second, the concept is applied in relation to a diverse set of stressors. A total of 153 (40.5%) studies referred to violence and conflict as predominant influences and drivers of fragility; of these studies, 56 (14.8%) studies specifically linked to the criteria of ‘fragile and conflict-affected states’ and a further 98 (25.9%) studies referred to security-related challenges, which destabilize state functions and directly result in the loss of life and well-being. However, 51 (13.5%) studies used fragility in relation to more chronic stressors—economic, political, social or environmental challenges—which had not yet compromised security. A further 173 (45.9%) studies referenced not overarching circumstances but rather specific conditions of fragility, e.g. as they may relate to challenges in offering personalized care to particularly vulnerable patients.

Third, in considering the impact of fragility on health, the concept is used in relation to various foci (hereafter called referents), typically health system functions, population capacities or the interface between these (e.g. in relation to the dynamic interaction between systems and communities in shaping health-seeking behaviour). Accordingly, for each reviewed document, we identified the primary referent to which fragility was related. The majority of documents (*n* = 159, 42.1%) focused on the concept in relation to health systems and their function, while 122 (32.3%) documents focused on outlining context-specific factors compromising health. A further 42 (11.1%) documents referred to fragile populations and 54 (14.3%) documents referred to fragility in the health system and community interface.

### Narrative synthesis: identifying attributes of fragility across literature referencing existing definitions

Only 56 (14.6%) studies provided explicit definitions of fragility. The most common anchor for such definitions was the concept of ‘fragile states’, although increasing reference to ‘fragile settings’ and ‘fragile and conflict-affected situations’ indicates a broadening use over time with an emphasis on the destabilizing influences of violence and conflict. Fragile states are generally seen as those where governments are unwilling or unable to deliver core functions and basic security to their people. Newbrander *et al.* (SR88) suggests the lack of governmental legitimacy and effectiveness as two defining characteristics of fragility at state level. Authors within this body of literature most frequently draw upon definitions offered by development institutions, e.g.:

*(a country) facing particularly severe development challenges such as weak institutional capacity, poor governance, political instability, and frequently ongoing violence or the legacy effects of past severe conflict* [International Development Association (2007) in SR11].

*when states lack political will and/or capacity to provide the basic functions needed for poverty reduction, development and to safeguard the security and human rights of their populations* [OECD (2007) in SR12].

*those (states) where the government cannot or will not deliver core functions to the majority of its people, including the poor* (SR317).

Haar and Rubenstein (SR151) note debates around assigning a rank or level to a state’s fragility, observing that it can be controversial to apply this term to some over others. Some have suggested that fragility is better viewed as a fluid concept that can decline or stabilize. Newbrander *et al.* (SR88) argue that state fragility exists alongside a continuum ranging from severe (where states are dependent on external assistance) to ready to drive development independently.

In addition to the emphasis on governance, mention of conflict as a precipitating factor appeared across several definitions and again linked to fluctuating degrees of fragility. Salama *et al.* (SR234) note United States Agency for International Development’s definition of fragility, which classifies fragile states in terms of ‘post-conflict, early recovery, arrested development, or deteriorating governance’. Gruber (SR328) differentiates conflict-affected fragile states from non-conflict fragile states, describing the latter as either those that have already experienced and moved away from conflict or those that are in relative situations of peace and stability yet still experience failures in basic service provision, security and systems of governance.

When discussing the way in which fragility impacts population health, authors across this body of literature typically refer to the significant difficulty health systems experience in responding and adapting to stressors and shocks. Newbrander *et al.* (SR88) offer a comprehensive synthesis of work in this vein, detailing how characteristics of country-level fragility such as prolonged conflict and exposure to violence result in health system-related ‘deficiencies’. Examples include health systems with insufficient service monitoring and co-ordination capacity or limited policy and information gathering mechanisms. These deficiencies contribute to an inability to provide sufficient and equitable health services to a population, resulting in lives lost. Globally, over a third of maternal deaths and half of deaths in children younger than 5 years occur within fragile states (Newbrander *et al.*, SR88).

### Narrative synthesis: identifying implicit understandings of fragility

The substantial majority—321 (85.1%)—of included studies, however, did not refer to a specific definition of fragility. Table 3 summarizes the application of the concept of fragility across these studies, using the three dimensions of level, stressor and referent noted earlier.

**Table 3:** Characterization of how fragility has been implicitly applied in the global health literature (*n* = 321)

Level	Stressor	Referent
State or regional level circumstances (*n* = 148), which affect population function	Security: violence and conflict as primary influences (*n* = 97)	Applications referring to setting characteristics and stressors (*n* = 45)
		Fragile health system (*n* = 41)
		Fragile population (*n* = 5)
		Fragile health system–population interface (*n* = 6)
	Chronic challenges: repeated social, political, economic and environmental stressors (which have not yet resulted in security challenges) (*n* = 51)	Applications referring to setting characteristics and stressors (*n* = 12)
		Fragile health system (*n* = 22)
		Fragile population (*n* = 10)
		Fragile health system–population interface (*n* = 7)
Within-country circumstances (*n* = 173)	Stressor not specified	Other application (*n* = 9)
		Fragile population (*n* = 27)
		Fragile health system (*n* = 96)
		Fragile health system–population interface (*n* = 41)

The discussion and five themes that follow are based on a comprehensive narrative synthesis of these studies (see Supplementary File 4). Within this synthesis, we focused on identifying in detail how the concept has been implicitly characterized and applied across each of the dimensions of level, stressor and referent. For health systems specifically, we additionally summarize how fragility is applied to each component of the health system.

#### Theme 1: when used in reference to security-related stressors, fragility focuses on health system functioning in a manner consistent with existing definitions

Across studies considering settings exposed to security-related challenges, the term is commonly applied similarly as within the literature referring to, and defining, ‘fragile and conflict-affected states’. For example, within those documents focusing on fragile settings or countries with current or past exposure of violence, fragility is used to indicate public health systems that have been severely fragmented or depleted due to the erosion of state-level capacities. For example, Muggah *et al.* (SR188) draw attention to Haiti’s characterization as a fragile, failing or failed state that experienced repeated bouts of violence and was further exposed to substantive exogenous shocks (earthquakes and hurricanes). Given such conditions, national and local health system resources were severely eroded—including destruction of infrastructure. The concomitant erosion of community resources is also frequently flagged, as is also emphasized in Newbrander *et al*. (SR88), and is additionally emphasized as a potential locus for health and wider programming. For example, Muggah *et al.* (SR188) note the importance of stabilization efforts that included both humanitarian health and peace-building interventions focusing on tackling the risk of violence via mediation and rebuilding health system capacity.

#### Theme 2: when used in relation to chronic stressors, fragility refers primarily to under-resourced and underperforming health systems

In contrast to the above, across those documents characterizing settings exposed to more diverse and chronic economic, sociopolitical and environmental stressors, fragility relates primarily to state and community systems functioning under chronic stress while still managing some basic service delivery and maintenance of health and well-being. Authors typically link fragility to cyclical poverty, social marginalization and extreme vulnerability to environmental conditions. Across this literature, e.g. Zaidi *et al.* (SR24) discuss how limited national financial resources and health system capacity resulted in contracting out primary maternal and child care services in Pakistan. While showing promise overall, the initiative had little success in reaching the rural poor, given historically low utilization in this disadvantaged population and increased costs of providing services in such areas. Similarly, Abubakari *et al.* (SR30) and Asokan (SR42) highlight how in northern Ghana and Nepal systems are yet to meet the needs of vulnerable groups. Although health systems here offer some basic services, decades of historical political neglect for remote country areas, along with limited financing and investment in human resources, mean that systems are not geared towards addressing the needs of high- and at-risk groups (e.g. mothers and children at risk in northern Ghana and groups vulnerable to recurrent natural disasters in Nepal).

#### Theme 3: financing and governance challenges are at the core of health system fragility

Across settings, when discussing health system functions, authors particularly emphasize the critical role of finances and governance, echoing a trend in the body of literature providing formal definitions of fragility. Specifically, authors discuss the scarcity of resources that many low- and middle-income countries report; this includes wider references to limited financial resources (SR34, 71, 68), limited transparency in the use of funds and resulting corruption (SR13, 71, 319) as well as dependence on external aid (SR79, 80, 97). Similarly, in relation to governance, authors note the varying degrees of governance capacity evident across the spectrum of settings under study. While, for settings exposed to conflict, authors discuss quasi-absent or corrupt and unaccountable national governance structures and the challenges of harmonizing international aid and donor initiatives (SR89, 147, 119, 326), challenges of health systems in otherwise stable settings are of a different nature. For example, difficulties in securing inter-sectoral collaboration and planning and further implementing integrated care initiatives are emphasized (SR81, 122, 289).

#### Theme 4: at the population level, fragility differs according to the stressor experienced

The reasons that populations are labelled as fragile or vulnerable differ according to the setting and stressor experienced. In settings exposed to security-related challenges, including violence and conflict, ethnic and political tension and circumstance are recognized as a primary influence for creating vulnerability—particularly around women and children (SR22, 5, 73, 82, 242). In contrast, across otherwise stable settings, poverty (SR 236, 332, 49), the inability to secure self-sufficiency due to limited education or training (SR18, 32, 44) and exposure to environmental risk due to poor housing are commonly seen as significant determinants of fragility (SR36, 131, 155). Vulnerable groups identified include persons affected by particularly debilitating illness (e.g. HIV patients, SR230, or those with comorbid conditions, SR139), the elderly and those of reduced mobility (SR285, 248) and socially marginalized populations (SR247, 142, 175).

#### Theme 5: across settings, fragility consistently references breakdowns at the interface between the community and the health system

A total of 54 (16.8%) documents use fragility in relation to the interaction of health systems and communities. In this study, the concept refers to barriers or breakdowns in the effective and legitimizing interaction between health systems (generally taken to mean public health systems) and the populations and communities they serve. Fragility is a concept that characterizes community and system interactions that are void of trust, stigmatizing, iniquitous, biased and reinforcing of traditional patient-provider power imbalances.

Two issues are at the core of such fragile interaction. First, health systems may not be prepared or equipped to acknowledge and address historical, political and personal circumstances when designing and delivering services. For example, health care providers who are not trained in chronic service delivery or patient communication may be ill-equipped to deal with a patient’s episodes of debilitating chronic illness and need for palliation at the end of life; providers may also fail to recognize the need of patients’ families for continued information and psychosocial support (SR135). Similarly, care providers who have experienced ethnic conflict may not be ready to deliver services to patients of other ethnicities and/or acknowledge collective emotional hurt (SR21).

Second, health system and community interaction may be labelled as fragile when services are not designed with local cultural norms in mind—as is the case when delivering family planning services to communities that prize large families (SR261), communities where health behaviours are anchored in strong ethnic identities (SR297) or in settings where female autonomy is limited (SR62). Of further relevance are instances where communities experience poor or negative care—e.g. due to stigmatization in the case of mental health conditions (SR4, 9). Such mismatches in service design, delivery and interaction with communities cause friction and ultimately undermine the confidence that local communities have in services; in turn, this exacerbates community-system tensions over time and leads to limited utilization of services.

Engagement and empowerment of local communities are generally cited as the main mechanisms to ensure appropriate tailoring of services to both cultural norms and redressing existing imbalances in power dynamics between care providers and patients or lapses in trust between institutions and care seekers. However, few suggestions for how to achieve engagement and empowerment are provided across the literature. The establishment of dialogue spaces and mechanisms for voicing complaints relating to health services and associated complaint resolution mechanisms (SR13), as well as establishment of mixed community/service delivery networks of care are amongst the few strategies noted (SR260, 281, 294).

## Discussion

This is the first scoping review to specifically focus on the concept of fragility as it relates to health. We acknowledge several limitations that require our conclusions to be considered with due caution. First, our intentionally broad search resulted in a large number of results and high heterogeneity in included studies. Double screening of articles and double extraction of data was not feasible given this volume of material. Double screening of a random 10% of search results and data extraction sheets from all reviewers being checked for quality and consistency by a second researcher were means of mitigation of risks of bias. Second, our search strategy and inclusion criteria may have missed some relevant literature as fragility is often referred to implicitly. To focus our review, we only included studies that explicitly used the term in the abstract or executive summary. However, analyses indicated a saturation of the concept of fragility and we, therefore, believe that any missing literature is unlikely to significantly alter findings.

Analyses identify an exponential growth in the use of the term in recent years and illustrate that initial and focused uses of the term—relating to ‘fragile states’ and/or ‘fragile and conflict-affected states’—have been superseded by far broader applications. Of the documents reviewed in this study, only 26.5% (100) of studies specifically refer to fragile states or contexts. A total of 173 (45.8%) documents do not refer to state-level fragility at all and instead discuss fragility in relation to population and/or health system functions.

Given such heterogeneous applications, what should we take the term ‘fragility’ to mean? First, we note that the global health literature portrays fragility as a multi-level concept. While the term is applied to state-specific circumstances, used predominantly to refer to fragile states or settings, fragility is also used to refer to health systems that have deficiencies or are otherwise under-resourced or underperforming. Furthermore, fragility may be used to describe specific communities and populations that are vulnerable.

Second, as noted through recent evolution of the OECD States of Fragility (OECD, 2018) framework, multiple dimensions of fragility are evident. We document applications of the term in relation to a range of stressors. While this includes prominent references to conflict and violence, increasingly chronic political, economic, social and environmental challenges are considered of relevance. Such precarious circumstances are additionally recognized as potential precipitators of conflict and violence or–conversely–as consequences thereof, highlighting the potentially cyclical nature of fragility.

Third, of relevance to global health specifically, we identify an emerging use of fragility in relation to health system–community relationships. Fragility is used as a concept that characterizes the breakdowns in trust between communities and health systems, with critical implications for health seeking. From the populations’ perspective, trust may be compromised due to the inequitable and inefficient delivery of care by the health system and/or inability of the system to adequately cater to complex health needs of vulnerable populations. From the systems’ perspective, trust may be compromised by health workers themselves in situations where the goals and emphases of service delivery do not align with local cultural norms.

While all three of these observations have implications for the use of the concept of fragility as an analytic framework in the field of global health, it is this final theme that has the clearest implication for intervention framing. Acknowledging the interface between health systems and communities to be a critical focus of fragility has particular significance, given that effective, accountable and legitimizing interactions between systems and communities are now recognized as key aspects of high-quality health systems (Kruk *et al.*, 2018). Furthermore, at this community–health system interface, this review highlights trust as a critical determinant of health seeking. This is consistent with wider emerging literature on health systems resilience and trust (Kittelsen and Keating, 2019; Woskie and Fallah, 2019), which also notes the significant role of the latter in mitigating the effects of emergencies.

## Conclusions

Fragility has increasingly been drawn upon as a concept to describe circumstances where it is challenging to drive advances in, or even maintain, population health. This review suggests that the diverse uses of the concept can be understood through a framing that distinguishes the principal level of analysis addressed, the major stressors considered and the specific focus (or referent) proposed. Across the wide literature ordered by this framing, the following five major themes can be identified: health systems functioning in the face of security-related stressors, under-resourced and underperforming health systems facing chronic stressors, health systems facing specific financing and governance challenges in contexts of fragility, context-specific sources of population fragility and breakdowns at the interface between the community and the health system. While sharing some features, each of these issues warrants discrete analysis and bears distinctive implications for intervention framing.

## Supplementary Material

czz142_Supplementary_Data
