# Supplementary material for: Understanding fragility: implications for global health research and practice
Source: Health Policy Plan. 2019 Dec 10;35(2):235–43. doi: 10.1093/heapol/czz142 (PMC7050687; doi:10.1093/heapol/czz142)
Supplement: czz142_Supplementary_Data [file czz142_supplementary_data.zip › czz142-suppl_data/02_Supplementary File 1 - Search strategies_no change.docx]

**Appendix 1: Search strategies**

## **Search strategy by source**

### ***Medline***

1. Health
2. (MH "Health Policy+") OR (MH "Health") OR (MH "Health Planning+") OR (MH "Health Manpower") OR (MH "Health Services") OR (MH "Health Care Economics and Organizations+") OR (MH "Health Care Reform")
3. TI Fragil* OR AB Fragil*
4. S1 AND S3
5. S2 AND S3
6. S4 OR S5
7. Osteoporosis
8. "fragile bone*"
9. "fragil* fracture*"
10. "Fragile X"
11. "medically fragile"
12. "fragile infant*"
13. "fragile site*"
14. "fragile histidine"
15. "bone fragility"
16. "fragile families"
17. "fragile patient*"
18. "fragile elder*"
19. "fragility index"
20. "fragile skin"
21. Fragilis
22. "osmotic fragility"
23. "tissue fragility"
24. "skin fragility"
25. S7 OR S8 OR S9 OR S10 OR S11 OR S12 OR S13 OR S14 OR S15 OR S16 OR S17 OR S18 OR S19 OR S20 OR S21 OR S22 OR S23 OR S24
26. S6 NOT S25

### ***CINAHL***

1. health
2. (MH "Health") OR (MH "Health Services Needs and Demand+") OR (MH "Health and Welfare Planning") OR (MH "Health Services Administration") OR (MH "Health Facilities+") OR (MH "Health Manpower") OR (MH "Health Services")
3. TI Fragil* OR AB Fragil*
4. S1 AND S3
5. S2 AND S3
6. S4 OR S5
7. Osteoporosis
8. "fragile bone*"
9. "fragil* fracture*"
10. "Fragile X"
11. "medically fragile"
12. "fragile infant*"
13. "fragile site*"
14. "fragile histidine"
15. "bone fragility"
16. "fragile families"
17. "fragile patient*"
18. "fragile elder*"
19. "fragility index"
20. "fragile skin"
21. Fragilis
22. "osmotic fragility"
23. "tissue fragility"
24. "skin fragility"
25. S7 OR S8 OR S9 OR S10 OR S11 OR S12 OR S13 OR S14 OR S15 OR S16 OR S17 OR S18 OR S19 OR S20 OR S21 OR S22 OR S23 OR S24
26. S6 NOT S25

### ***Global Health Library***

health AND (fragile OR fragility) AND NOT (osteoporosis OR "fragile bones" OR "fragility fracture" OR "fragile X" OR "medically fragile" OR "fragile infant" OR "fragile infants" OR "fragile site" OR "fragile sites" OR "fragile histidine" OR "bone fragility" OR "fragile families" OR "fragile patients" OR "fragile elderly" OR "fragility index" OR "fragile skin" OR fragilis OR "osmotic fragility" OR "tissue fragility" OR "skin fragility")

Exc. Medline (i.e. LILACS (Americas); WPRIM (Western Pacific); IMEMR (Eastern Mediterranean); AIM (Africa); BBO - dentistry (Brazil); IMSEAR (South-EastAsia); WHOLIS (KMS))

## **Grey Literature and Journals:**

***Search Strategy A***

Health AND (fragile OR fragility)

***Search Strategy B***

health AND (fragile OR fragility) AND NOT (osteoporosis OR "fragile bones" OR "fragility fracture" OR "fragile X" OR "medically fragile" OR "fragile infant" OR "fragile infants" OR "fragile site" OR "fragile sites" OR "fragile histidine" OR "bone fragility" OR "fragile families" OR "fragile patients" OR "fragile elderly" OR "fragility index" OR "fragile skin" OR fragilis OR "osmotic fragility" OR "tissue fragility" OR "skin fragility")

***Strategy C***

Search 1: health AND fragile

Search 2: health AND fragility

***Strategy D***

Health AND fragile

Filter: issue date equals [2010-2017]

**Search Strategies used for grey literature and journal searches**

| **Source** | **Search Strategy** |
| --- | --- |
| ***Grey Literature*** |  |
| Health Systems Global | A |
| OpenGrey | B |
| Grey Literature Report | A |
| Management Sciences for Health (MSH) | A |
| Department for International Development | A |
| The World Bank | C |
| WHO IRIS | D |
|  |  |
| ***Journals*** |  |
| Conflict and Health | A |
| Health Research Policy and Systems | A |
| Health Policy and Planning | A |
| Global Health: Science and Practice | C |
| Social Science and Medicine | A |
