# Supplementary material for: Understanding fragility: implications for global health research and practice
Source: Health Policy Plan. 2019 Dec 10;35(2):235–43. doi: 10.1093/heapol/czz142 (PMC7050687; doi:10.1093/heapol/czz142)
Supplement: czz142_Supplementary_Data [file czz142_supplementary_data.zip › czz142-suppl_data/02_Supplementary File 2 - Data extraction template_no change.docx]

**Appendix 2: Data extraction template**

| **Domain** | **Extraction Item** | **Instruction** | **Example (fictional)** |
| --- | --- | --- | --- |
| **Identifiers** | First author | Note surname, first initial of the first author | Author, I. |
|  | Other authors | Note surname, first initial of remaining authors separated by ; | Author 2, I.; Author 3, I. |
|  | Year | Note year of publication | 2017 |
|  | Title | Note title of publication | Health system fragility: findings of a scoping review |
|  | Author affiliations | Note author affiliations listed in publication | Queen Margaret University |
| **Primary descripts** | Country or setting | Note the country and/or specific setting studied | Lebanon: North Bekaa area |
|  | Context | Note key words relating to the study context | Time of the Syria conflict, regional instability, displacement into Bekaa valley |
|  | Purpose of study | Extract a quote (or paraphrase main points) summarizing study purpose | This study aims to describe displacement of Syrian refugees into the Bekaa valley in Lebanon and document ancillary challenges in health service delivery |
|  | Dates of Study | Dates over which study was carried out | Stakeholder interviews carried out from 2011-2013 |
| **Methodology** | Study design | Extract information on broad study design: e.g. expertimental study (i.e. an intervention was tried out) or otherwise; please note the design used | Non-experimental study: qualitative design |
|  | Interventions/policies of note | Extract information on the intervention/policy mentioned | E.g. nutrition intervention to address malnutrition among refugee and host populations |
|  | Key population mentioned | If population groups are mentioned, please note which groups are mentioned | Refugees from Syria |
|  | Methods | Please note the exact methods by which the study was carried out | Semi-structured interviews with key stakeholders (23 people interviewed) |
| **Findings** | Findings/important results | Please extract information on main findings of study; may be extracted using summary bullet points or if relevant direct quotation | Displacement into region severe during 2011/12; - Nutritional status among those arriving was specifically poor |
| **Limitations and place in literature** | Limitations - author specified | Note any study limitations raised by authors | Not generalizable to experience of refugees in other areas of Lebanon |
|  | Limitations - reviewer note | Note any study limitations you believe applicable | Small interview sample |
|  | Study value | Note the value of the study/place in the literature as proposed by the authors | Exploring experience of specific refugee sub-population |
|  | Research gaps remaining | If authors mention research gaps to be addressed, please note them (quotation) | Further studies into other populations recommended |
|  | Translation into practice | If authors mention how their findings were taken up into practice (e.g. policy), please note this | Informed policy by Lebanon Ministry of Health |
| **Fragility** | Primary concept referenced | Note how the word 'fragile'/'fragility' is used (e.g. is it in relation to a setting, a situation, a population, a system, all of the aforementioned?) | Fragile state and fragile population |
|  | Quotation | Extract the main quotation/s where the term is used. | In the region, Syria is labelled as a fragile state (World Bank, 2012) |
|  | Definition | Is an explicit definition/conceptualization of the term included? If so, please extract all quotations relating to this here | NA |
|  | Sources referenced | When the term is used, do the authors refer to other sources? If so, please extract references | World Bank 2012 - Reference X |
|  | Determinants | Are there any issues in relation to fragility that the authors highlight as causative? Please extract a quotation if possible | Poverty and political instability are primary contributors to fragility |
