# Supplementary material for: Understanding fragility: implications for global health research and practice
Source: Health Policy Plan. 2019 Dec 10;35(2):235–43. doi: 10.1093/heapol/czz142 (PMC7050687; doi:10.1093/heapol/czz142)
Supplement: czz142_Supplementary_Data [file czz142_supplementary_data.zip › czz142-suppl_data/02_Supplementary File 3 - Reference list of included studies_edited_final.docx]

**List of Included Studies with Systematic Review Index Number**

| **SR** | **Reference** |
| --- | --- |
| 1 | Bourdeaux, M. et al., 2015. A cross-case comparative analysis of international security forces’ impacts on health systems in conflict-affected and fragile states. *Conflict And Health*, 9, p.14. Available at: http://search.ebscohost.com/login.aspx?direct=true&db=cmedm&AN=25878724&site=ehost-live. |
| 2 | Siriwardhana, C. et al. (2014). A systematic review of resilience and mental health outcomes of conflict-driven adult forced migrants. *Conflict and Health*. 8:13. Available from: https://doi.org/10.1186/1752-1505-8-13 |
| 3 | Bertone, M.P. et al. (2014). A window of opportunity for reform in post-conflict settings? The case of Human Resources for Health policies in Sierra Leone, 2002–2012. *Conflict and Health*. 8:11. Available from: https://doi.org/10.1186/1752-1505-8-11 |
| 4 | Chynoweth, S.K. (2015). Advancing reproductive health on the humanitarian agenda: the 2012-2014 global review. *Conflict and Health*. 9 (Suppl 1) :I1. Available from: https://doi.org/10.1186/1752-1505-9-S1-I1 |
| 5 | Muldoon, K.A. et al. (2014). After abduction: exploring access to reintegration programs and mental health status among young female abductees in Northern Uganda. *Conflict and Health*. 8:5. Available from: https://doi.org/10.1186/1752-1505-8-5 |
| 6 | Nickerson, J.W. et al. (2015). Assessments of health services availability in humanitarian emergencies: a review of assessments in Haiti and Sudan using a health systems approach. *Conflict and Health*. 9:20. Available from: https://doi.org/10.1186/s13031-015-0045-6 |
| 7 | Faqir, M. et al. (2015). Availability and distribution of human resources for provision of comprehensive emergency obstetric and newborn care in Afghanistan: a cross-sectional study. *Conflict and Health*. 9:9 Available from: https://doi.org/10.1186/s13031-015-0037-6 |
| 8 | Calain, P. & Abu Sa’Da, C., 2015. Coincident polio and Ebola crises expose similar fault lines in the current global health regime. *Conflict And Health*, 9, p.29. Available at: http://search.ebscohost.com/login.aspx?direct=true&db=cmedm&AN=26380580&site=ehost-live. |
| 9 | Trani, J-F. et al. (2016). Community based system dynamic as an approach for understanding and acting on messy problems: a case study for global mental health intervention in Afghanistan. *Conflict and Health*. 10:25. Available from: https://doi.org/10.1186/s13031-016-0089-2 |
| 10 | Najafizada, S.A.M. et al. (2014). Community health workers of Afghanistan: a qualitative study of a national program. *Conflict and Health*. 8:26. Available from: https://doi.org/10.1186/1752-1505-8-26 |
| 11 | Bruckner, C. & Checchi, F., 2011. Detection of infectious disease outbreaks in twenty-two fragile states, 2000-2010: a systematic review. *Conflict And Health*, 5, p.13. Available at: http://search.ebscohost.com/login.aspx?direct=true&db=cmedm&AN=21861869&site=ehost-live. |
| 12 | McPake, B. et al. (2015). Ebola in the context of conflict affected states and health systems: case studies of Northern Uganda and Sierra Leone. *Conflict and Health*. 9:23 Available from: https://doi.org/10.1186/s13031-015-0052-7 |
| 13 | Ho, L.S. et al. (2015). Effects of a community scorecard on improving the local health system in Eastern Democratic Republic of Congo: qualitative evidence using the most significant change technique. *Conflict and Health*. 9:27 Available from: https://doi.org/10.1186/s13031-015-0055-4 |
| 14 | Rahimzai, M. et al. (2014). Engaging frontline health providers in improving the quality of health care using facility-based improvement collaboratives in Afghanistan: case study. *Conflict and Health*. 8:21. Available from: https://doi.org/10.1186/1752-1505-8-21 |
| 15 | Witter, S. et al. (2016). Evolution of policies on human resources for health: opportunities and constraints in four post-conflict and post-crisis settings. *Conflict and Health*. 10:31. Available from: https://doi.org/10.1186/s13031-016-0099-0 |
| 16 | Cetorelli, V. & Shabila, N.P., 2014. Expansion of health facilities in Iraq a decade after the US-led invasion, 2003-2012. *Conflict And Health*, 8, p.16. Available at: http://search.ebscohost.com/login.aspx?direct=true&db=cmedm&AN=25221620&site=ehost-live. |
| 17 | Patel, P. et al. (2015). Exploring the influence of the Global Fund and the GAVI Alliance on health systems in conflict-affected countries. *Conflict and Health*. 9:7. Available from: https://doi.org/10.1186/s13031-015-0031-z |
| 18 | Tanabe, M. et al. (2017). Family planning in refugee settings: findings and actions from a multi-country study. *Conflict and Health*. 11:9. Available from: https://doi.org/10.1186/s13031-017-0112-2 |
| 19 | Raven, J. et al. (2014). Fragile and conflict affected states: report from the Consultation on Collaboration for Applied Health Research and Delivery. *Conflict and Health*. 8:15. Available from: https://doi.org/10.1186/1752-1505-8-15 |
| 20 | Casey, S.E. et al. (2015). Progress and gaps in reproductive health services in three humanitarian settings: mixed-methods case studies. *Conflict and Health*. 9 (Suppl 1): S3. Available from: https://doi.org/10.1186/1752-1505-9-S1-S3 |
| 21 | Wang, S-J. et al. (2012). Survivors of war in northern Kosovo (III): The role of anger and hatred in pain and PTSD and their interactive effects on career outcome, quality of sleep and suicide ideation. *Conflict and Health*. 6:4. Available from: https://doi.org/10.1186/1752-1505-6-4 |
| 22 | Hutchinson, A. et al. (2017). Understanding processes of risk and protection that shape the sexual and reproductive health of young women affected by conflict: the price of protection. *Conflict and Health*.11:15 Available from: https://doi.org/10.1186/s13031-017-0117-x |
| 23 | Kawonga, M. et al. (2012). Aligning vertical interventions to health systems: a case study of the HIV monitoring and evaluation system in South Africa. *Health Research Policy and Systems*. 10:2Available from: https://doi.org/10.1186/1478-4505-10-2 |
| 24 | Zaidi, S. et al. (2015). Can contracted out health facilities improve access, equity, and quality of maternal and newborn health services? Evidence from Pakistan. *Health Research Policy and Systems*. 13 (Suppl 1):S54. Available from: https://doi.org/10.1186/s12961-015-0041-8 |
| 25 | Parker, M. & Allen, T. (2011). Does mass drug administration for the integrated treatment of neglected tropical diseases really work? Assessing evidence for the control of schistosomiasis and soil-transmitted helminths in Uganda. *Health Research Policy and Systems*. 9:3 Available from: https://doi.org/10.1186/1478-4505-9-3 |
| 26 | Sombié, I. et al. (2017). Evaluation of regional project to strengthen national health research systems in four countries in West Africa: lessons learned. *Health Research Policy and Systems. 15 (Suppl 1):46. Available from: https://doi.org/10.1186/s12961-017-0214-8* |
| 27 | Woodward, A. et al. (2017). Health systems research in fragile and conflict affected states: a qualitative study of associated challenges. *Health Research Policy and Systems*. 15:44.Available from: https://doi.org/10.1186/s12961-017-0204-x |
| 28 | Martins, J.S. et al.(2009). Malaria control in Timor-Leste during a period of political instability: what lessons can be learned?. *Conflict and Health*. 3:11. Available from: https://doi.org/10.1186/1752-1505-3-11 |
| 29 | Abu-Zaineh, M. et al., 2011. Measuring and decomposing socioeconomic inequality in healthcare delivery: A microsimulation approach with application to the Palestinian conflict-affected fragile setting. *Social Science & Medicine (1982)*, 72(2), pp.133–141. Available at: http://search.ebscohost.com/login.aspx?direct=true&db=cmedm&AN=21145153&site=ehost-live. |
| 30 | Abubakari, A., Kynast-Wolf, G. & Jahn, A., 2015. Prevalence of abnormal birth weight and related factors in Northern region, Ghana. *BMC Pregnancy And Childbirth*, 15, p.335. Available at: http://search.ebscohost.com/login.aspx?direct=true&db=cmedm&AN=26670886&site=ehost-live. |
| 31 | Agyepong, I.A. et al., 2015. Health Policy, Health Systems Research and Analysis Capacity Assessment of the School of Public Health, University of Ghana. *Ghana Medical Journal*, 49(3), pp.200–213. Available at: http://search.ebscohost.com/login.aspx?direct=true&db=cmedm&AN=26693197&site=ehost-live. |
| 32 | Ahmad, K. et al., 2015. Self-perceived barriers to eye care in a hard-to-reach population: the Karachi Marine Fishing Communities Eye and General Health Survey. *Investigative Ophthalmology & Visual Science*, 56(2), pp.1023–1032. Available at: http://search.ebscohost.com/login.aspx?direct=true&db=cmedm&AN=25564446&site=ehost-live. |
| 33 | Ahmadzai, H. et al., 2008. Scaling up TB DOTS in a fragile state: post-conflict Afghanistan. *The International Journal Of Tuberculosis And Lung Disease: The Official Journal Of The International Union Against Tuberculosis And Lung Disease*, 12(2), pp.180–185. Available at: http://search.ebscohost.com/login.aspx?direct=true&db=cmedm&AN=18230251&site=ehost-live. |
| 34 | Al Hilfi, T.K.Y., 2014. Toward a healthier Iraq. *The Yale Journal Of Biology And Medicine*, 87(3), pp.289–297. Available at: http://search.ebscohost.com/login.aspx?direct=true&db=cmedm&AN=25191144&site=ehost-live. |
| 35 | Alam, K. & Oliveras, E., 2014. Retention of female volunteer community health workers in Dhaka urban slums: a prospective cohort study. *Human Resources For Health*, 12, p.29. Available at: http://search.ebscohost.com/login.aspx?direct=true&db=cmedm&AN=24886046&site=ehost-live. |
| 36 | Alecrim, T.F. de A. et al., 2016. Experience of health professionals in care of the homeless population with tuberculosis. *Revista Da Escola De Enfermagem Da U S P*, 50(5), pp.808–815. Available at: http://search.ebscohost.com/login.aspx?direct=true&db=cmedm&AN=27982400&site=ehost-live. |
| 37 | Alemnji, G.A. et al., 2014. Strengthening national health laboratories in sub-Saharan Africa: a decade of remarkable progress. *Tropical Medicine & International Health: TM & IH*, 19(4), pp.450–458. Available at: http://search.ebscohost.com/login.aspx?direct=true&db=cmedm&AN=24506521&site=ehost-live. |
| 38 | Alemnji, G.A. et al., 2012. Strengthening national laboratory health systems in the Caribbean Region. *Global Public Health*, 7(6), pp.648–660. Available at: http://search.ebscohost.com/login.aspx?direct=true&db=cmedm&AN=22519703&site=ehost-live. |
| 39 | Alonso, W.J. et al., 2015. Beyond crystal balls: crosscutting solutions in global health to prepare for an unpredictable future. *BMC Public Health*, 15, p.955. Available at: http://search.ebscohost.com/login.aspx?direct=true&db=cmedm&AN=26400682&site=ehost-live. |
| 40 | Amde, W.K., Sanders, D. & Lehmann, U., 2014. Building capacity to develop an African teaching platform on health workforce development: a collaborative initiative of universities from four sub Saharan countries. *Human Resources For Health*, 12, p.31. Available at: http://search.ebscohost.com/login.aspx?direct=true&db=cmedm&AN=24886267&site=ehost-live. |
| 41 | Arya, S.C. & Agarwal, N., 2012. Quality control inclinical diagnostic laboratories in remote and rural areas in Africa. *The Pan African Medical Journal*, 13, p.76. Available at: http://search.ebscohost.com/login.aspx?direct=true&db=cmedm&AN=23396956&site=ehost-live. |
| 42 | Asokan, G. V & Vanitha, A., 2017. Disaster response under One Health in the aftermath of Nepal earthquake, 2015. *Journal Of Epidemiology And Global Health*, 7(1), pp.91–96. Available at: http://search.ebscohost.com/login.aspx?direct=true&db=cmedm&AN=27059251&site=ehost-live. |
| 43 | Atti, E. & Gulis, G., 2017. Political determinants of progress in the MDGs in Sub-Saharan Africa. *Global Public Health*, 12(11), pp.1351–1368. Available at: http://search.ebscohost.com/login.aspx?direct=true&db=cmedm&AN=27166318&site=ehost-live. |
| 44 | Aved, B.M. et al., 1993. Barriers to prenatal care for low-income women. *The Western Journal Of Medicine*, 158(5), pp.493–498. Available at: http://search.ebscohost.com/login.aspx?direct=true&db=cmedm&AN=8342265&site=ehost-live. |
| 45 | Axelsson, R. & Axelsson, S.B., 2006. Integration and collaboration in public health--a conceptual framework. *The International Journal Of Health Planning And Management*, 21(1), pp.75–88. Available at: http://search.ebscohost.com/login.aspx?direct=true&db=cmedm&AN=16604850&site=ehost-live. |
| 46 | Bagcchi, S., 2016. Cholera in Iraq strains the fragile state. *The Lancet. Infectious Diseases*, 16(1), pp.24–25. Available at: http://search.ebscohost.com/login.aspx?direct=true&db=cmedm&AN=26738834&site=ehost-live. |
| 47 | Barak, Y. & Cohen, A., 2003. Characterizing the elderly homeless: a 10-year study in Israel. *Archives Of Gerontology And Geriatrics*, 37(2), pp.147–155. Available at: http://search.ebscohost.com/login.aspx?direct=true&db=cmedm&AN=12888228&site=ehost-live. |
| 48 | Barennes, H. et al., 2015. Evidence of High Out of Pocket Spending for HIV Care Leading to Catastrophic Expenditure for Affected Patients in Lao People’s Democratic Republic. *Plos One*, 10(9), pp.e0136664–e0136664. Available at: http://search.ebscohost.com/login.aspx?direct=true&db=cmedm&AN=26327558&site=ehost-live. |
| 49 | Behets, F. et al., 2009. Reducing vertical HIV transmission in Kinshasa, Democratic Republic of Congo: trends in HIV prevalence and service delivery. *AIDS Care*, 21(5), pp.583–590. Available at: http://search.ebscohost.com/login.aspx?direct=true&db=cmedm&AN=19444666&site=ehost-live. |
| 50 | Béland, F. et al., 2006. Integrated services for frail elders (SIPA): a trial of a model for Canada. *Canadian Journal On Aging = La Revue Canadienne Du Vieillissement*, 25(1), pp.5–42. Available at: http://search.ebscohost.com/login.aspx?direct=true&db=cmedm&AN=16770746&site=ehost-live. |
| 51 | Benatar, S.R., 2013. Global health, vulnerable populations, and law. *The Journal Of Law, Medicine & Ethics: A Journal Of The American Society Of Law, Medicine & Ethics*, 41(1), pp.42–47. Available at: http://search.ebscohost.com/login.aspx?direct=true&db=cmedm&AN=23581656&site=ehost-live. |
| 52 | Bennett, S. et al., 2012. Approaches to developing the capacity of health policy analysis institutes: a comparative case study. *Health Research Policy And Systems*, 10, p.7. Available at: http://search.ebscohost.com/login.aspx?direct=true&db=cmedm&AN=22390185&site=ehost-live. |
| 53 | Berendes, S. et al., 2014. Assessing the quality of care in a new nation: South Sudan’s first national health facility assessment. *Tropical Medicine & International Health: TM & IH*, 19(10), pp.1237–1248. Available at: http://search.ebscohost.com/login.aspx?direct=true&db=cmedm&AN=25134414&site=ehost-live. |
| 54 | Bernays, S., Rhodes, T. & Barnett, T., 2007. Hope: a new way to look at the HIV epidemic. *AIDS (London, England)*, 21 Suppl 5, pp.S5–S11. Available at: http://search.ebscohost.com/login.aspx?direct=true&db=cmedm&AN=18090269&site=ehost-live. |
| 55 | Berti, H.W. et al., 2008. Movement undertaken by newly graduated nurses towards the strengthening of their professional autonomy and towards patient autonomy. *Revista Latino-Americana De Enfermagem*, 16(2), pp.184–191. Available at: http://search.ebscohost.com/login.aspx?direct=true&db=cmedm&AN=18506335&site=ehost-live. |
| 56 | Bertone, M.P., Lurton, G. & Mutombo, P.B., 2016. Investigating the remuneration of health workers in the DR Congo: implications for the health workforce and the health system in a fragile setting. *Health Policy And Planning*, 31(9), pp.1143–1151. Available at: http://search.ebscohost.com/login.aspx?direct=true&db=cmedm&AN=26758540&site=ehost-live. |
| 57 | Bhutta, Z.A. et al., 2013. Reproductive, maternal, newborn, and child health in Pakistan: challenges and opportunities. *Lancet (London, England)*, 381(9884), pp.2207–2218. Available at: http://search.ebscohost.com/login.aspx?direct=true&db=cmedm&AN=23684261&site=ehost-live. |
| 58 | Bicknell, W.J. & Parks, C.L., 1989. As children survive: dilemmas of aging in the developing world. *Social Science & Medicine (1982)*, 28(1), pp.59–67. Available at: http://search.ebscohost.com/login.aspx?direct=true&db=cmedm&AN=2648594&site=ehost-live. |
| 59 | Bisika, T., 2010. Health systems strengthening in conflict situations. *East African Journal Of Public Health*, 7(3), pp.277–281. Available at: http://search.ebscohost.com/login.aspx?direct=true&db=cmedm&AN=21516969&site=ehost-live. |
| 60 | Bizimana, J.-P., Twarabamenye, E. & Kienberger, S., 2015. Assessing the social vulnerability to malaria in Rwanda. *Malaria Journal*, 14, p.2. Available at: http://search.ebscohost.com/login.aspx?direct=true&db=cmedm&AN=25566988&site=ehost-live. |
| 61 | Blanchet, K. et al., 2014. Physical rehabilitation in post-conflict settings: analysis of public policy and stakeholder networks. *Disability And Rehabilitation*, 36(18), pp.1494–1501. Available at: http://search.ebscohost.com/login.aspx?direct=true&db=cmedm&AN=23672208&site=ehost-live. |
| 62 | Boddam-Whetham, L. et al., 2016. Vouchers in Fragile States: Reducing Barriers to Long-Acting Reversible Contraception in Yemen and Pakistan. *Global Health, Science And Practice*, 4 Suppl 2, pp.S94–S108. Available at: http://search.ebscohost.com/login.aspx?direct=true&db=cmedm&AN=27540129&site=ehost-live. |
| 63 | Boone, P. et al., 2016. Effects of community health interventions on under-5 mortality in rural Guinea-Bissau (EPICS): a cluster-randomised controlled trial. *The Lancet. Global Health*, 4(5), pp.e328–e335. Available at: http://search.ebscohost.com/login.aspx?direct=true&db=cmedm&AN=27102196&site=ehost-live. |
| 64 | Bornemisza, O. et al., 2010. Health Aid governance in fragile states: the global fund experience. *Glob Health Gov*, 4(1), p.[18]-[18]. Available at: http://ghgj.org/Lazarus_final.pdf. |
| 65 | Bornemisza, O. et al., 2010. Promoting health equity in conflict-affected fragile states. *Social Science & Medicine (1982)*, 70(1), pp.80–88. Available at: http://search.ebscohost.com/login.aspx?direct=true&db=cmedm&AN=19853342&site=ehost-live. |
| 66 | Both, J.M.C. & van Roosmalen, J., 2010. The impact of Prevention of Mother to Child Transmission (PMTCT) programmes on maternal health care in resource-poor settings: looking beyond the PMTCT programme--a systematic review. *BJOG: An International Journal Of Obstetrics And Gynaecology*, 117(12), pp.1444–1450. Available at: http://search.ebscohost.com/login.aspx?direct=true&db=cmedm&AN=20937071&site=ehost-live. |
| 67 | Bouder, F., 2015. Risk communication of vaccines: challenges in the post-trust environment. *Current Drug Safety*, 10(1), pp.9–15. Available at: http://search.ebscohost.com/login.aspx?direct=true&db=cmedm&AN=25859669&site=ehost-live. |
| 68 | Bousquat, A. et al., 2017. Primary health care and the coordination of care in health regions: managers’ and users’ perspective. *Ciencia & Saude Coletiva*, 22(4), pp.1141–1154. Available at: http://search.ebscohost.com/login.aspx?direct=true&db=cmedm&AN=28444041&site=ehost-live. |
| 69 | Stilwell, B. (2016). *A safe bet: investing in resilient health systems for everyone*. Available from: http://www.healthsystemsglobal.org/blog/140/A-safe-bet-investing-in-resilient-health-systems-for-everyone.html [Accessed 7th November 2018] |
| 70 | Commins, S. (2015). *‘Fragility, Conflict and Violence’ and health: New questions for research and policy design.* Available from: http://www.healthsystemsglobal.org/blog/56/-Fragility-Conflict-and-Violence-and-health-New-questions-for-research-and-policy-design.html [Accessed 7th November 2018] |
| 71 | Middle East Research Institute. (2015). *Health System Challenges in the face of the humanitarian crisis in Iraq*. Available from: http://www.healthsystemsglobal.org/upload/resource/Health_System_Challenges_in_the_face_of_the_humanitarian_crisis_in_Iraq___Stakeholder_Meeting_Report_-_.pdf [Accessed 7th November 2018] |
| 72 | Norris, J. (2015). *Fragile States Report*. Available from: http://www.savethechildren.org/atf/cf/%7B9def2ebe-10ae-432c-9bd0-df91d2eba74a%7D/FRAGILESTATES-REPORT_WEB.PDF [Accessed 16th November 2017] |
| 73 | Quinn, M (ed). (2016). *Governance and Health in Post-Conflict Countries: The Ebola Outbreak in Liberia and Sierra Leone*. New York: International Peace Institute. Available from: https://www.ipinst.org/wp-content/uploads/2016/06/1606_Governance-and-Health.pdf [Accessed 7th November 2018] |
| 74 | Management Sciences for Health. (2011). *Improving Health in Rwanda with Performance-Based Financing*. Available from: https://www.msh.org/news-events/stories/improving-health-in-rwanda-with-performance-based-financing [Accessed 7th November 2018] |
| 75 | Haar, R.J. & Rubenstein, L.S., 2012. Health in fragile and post-conflict states: a review of current understanding and challenges ahead. *Medicine, Conflict, And Survival*, 28(4), pp.289–316. Available at: http://search.ebscohost.com/login.aspx?direct=true&db=cmedm&AN=23421305&site=ehost-live. |
| 76 | Management Sciences for Health. (2014). *Health Workers, Patients Under Attack*. Available from: https://www.msh.org/news-events/press-room/health-workers-patients-under-attack [Accessed 7th November 2018] |
| 77 | Management Sciences for Health. (2008). *Community Health Workers in Afghanistan Bridge a Gap in Times of High Turnover*. Available from: https://www.msh.org/news-events/stories/community-health-workers-in-afghanistan-bridge-a-gap-in-times-of-high-turnover [Accessed 7th November 2018] |
| 78 | Management Sciences for Health. (n.d.). *Fragile States*. Available from: https://www.msh.org/our-work/practices/advocacy/fragile-states [Accessed 7th November 2018] |
| 79 | Quick, J.D. (2010). *Building Health Systems in Fragile States*. Available from: https://www.msh.org/blog/2010/10/29/building-health-systems-in-fragile-states [Accessed 7th November 2018] |
| 80 | Management Sciences for Health. (2008). *Closing the Gap by Strengthening Health Systems*. Available from: https://www.msh.org/news-events/stories/closing-the-gap-by-strengthening-health-systems [Accessed 7th November 2018] |
| 81 | Management Sciences for Health. (2008). *Managing Systems: Improving Use of Resources to Effectively Fight AIDS in Uganda*. Available from: https://www.msh.org/news-events/stories/managing-systems-improving-use-of-resources-to-effectively-fight-aids-in-uganda [Accessed 7th November 2018] |
| 82 | Quick, J.D. (2012). *President Obama: Forward on Development and Global Health*. Available from: https://www.msh.org/blog/2012/11/07/president-obama-forward-on-development-and-global-health [Accessed 7th November 2018] |
| 83 | Management Sciences for Health. (2010). *Improving Health Services in a Fragile State: Liberia*. Available from: https://www.msh.org/news-events/stories/improving-health-services-in-a-fragile-state-liberia [Accessed 7th November 2018] |
| 84 | Management Sciences for Health. (2012). *MSH's Working Principles for Health Development Initiatives in Fragile States*. Available from: https://www.msh.org/blog/2012/04/07/mshs-working-principles-for-health-development-initiatives-in-fragile-states [Accessed 7th November 2018] |
| 85 | USAID & Community-Based Support for Orphans and Vulnerable Children (CUBS). (2014). *Not Alone: Creating Resiliency in Children through Layered Support Systems*. Available from: https://www.msh.org/sites/msh.org/files/nigeria_cubseop_17junwebv.pdf [Accessed 7th November 2018] |
| 86 | Management Sciences for Health. (2011). *Integrating Health Services is the Means, Not the End*. Available from: https://www.msh.org/news-events/stories/integrating-health-services-is-the-means-not-the-end [Accessed 7th November 2018] |
| 87 | Management Sciences for Health. (n.d.). *Strengthening Health Systems in Fragile States*. Available from: https://www.msh.org/resources/strengthening-health-systems-in-fragile-states [Accessed 7th November 2018] |
| 88 | Newbrander, W. (2007). Rebuilding Health Systems and Providing Health Services in Fragile States. *MSH Occassional Paper.*7. Available from: https://www.msh.org/sites/msh.org/files/rebuilding-health-systems-and-providing-health-services-in-fragile-states.pdf [Accessed 7th November 2018] |
| 89 | Adano, U. & Nelson, D. (2015). *Lessons in Post-Conflict Recovery: Developing a Health Workforce in Afghanistan and South Sudan.* Available from: https://www.msh.org/blog/2015/02/27/lessons-in-post-conflict-recovery-developing-a-health-workforce-in-afghanistan-and [Accessed 7th November 2018] |
| 90 | Siu, G.E., Wight, D. & Seeley, J., 2012. How a masculine work ethic and economic circumstances affect uptake of HIV treatment: experiences of men from an artisanal gold mining community in rural eastern Uganda. *Journal Of The International AIDS Society*, 15 Suppl 1, pp.1–9. Available at: http://search.ebscohost.com/login.aspx?direct=true&db=cmedm&AN=22713356&site=ehost-live. |
| 91 | Smallwood, R., 2003. Safety and quality in healthcare: what can England and Australia learn from each other? *Clinical Medicine (London, England)*, 3(1), pp.68–73. Available at: http://search.ebscohost.com/login.aspx?direct=true&db=cmedm&AN=12617419&site=ehost-live. |
| 92 | Sousa, V.D., Ramalho, P.I. & Silveira, D., 2016. Sharing regulatory data as tools for strengthening health systems in the Region of the Americas. *Revista Panamericana De Salud Publica = Pan American Journal Of Public Health*, 39(5), pp.245–254. Available at: http://search.ebscohost.com/login.aspx?direct=true&db=cmedm&AN=27706398&site=ehost-live. |
| 93 | Ssonko, C. et al., 2017. Delivering HIV care in challenging operating environments: the MSF experience towards differentiated models of care for settings with multiple basic health care needs. *Journal Of The International AIDS Society*, 20(Suppl 4), pp.14–20. Available at: http://search.ebscohost.com/login.aspx?direct=true&db=cmedm&AN=28770590&site=ehost-live. |
| 94 | Steinhardt, L.C. & Peters, D.H., 2010. Targeting accuracy and impact of a community-identified waiver card scheme for primary care user fees in Afghanistan. *International Journal For Equity In Health*, 9, p.28. Available at: http://search.ebscohost.com/login.aspx?direct=true&db=cmedm&AN=21114851&site=ehost-live. |
| 95 | Sypek, S., Clugston, G. & Phillips, C., 2008. Critical health infrastructure for refugee resettlement in rural Australia: case study of four rural towns. *The Australian Journal Of Rural Health*, 16(6), pp.349–354. Available at: http://search.ebscohost.com/login.aspx?direct=true&db=cmedm&AN=19032207&site=ehost-live. |
| 96 | Tang, S.T. et al., 2016. Prevalence of severe depressive symptoms increases as death approaches and is associated with disease burden, tangible social support, and high self-perceived burden to others. *Supportive Care In Cancer: Official Journal Of The Multinational Association Of Supportive Care In Cancer*, 24(1), pp.83–91. Available at: http://search.ebscohost.com/login.aspx?direct=true&db=cmedm&AN=25933701&site=ehost-live. |
| 97 | Tappis, H. et al., 2016. Context matters: Successes and challenges of intrapartum care scale-up in four districts of Afghanistan. *Global Public Health*, 11(4), pp.387–406. Available at: http://search.ebscohost.com/login.aspx?direct=true&db=cmedm&AN=26645366&site=ehost-live. |
| 98 | Teixeira, M.R., Couto, M.C.V. & Delgado, P.G.G., 2017. Primary care and collaborative care in children and adolescents psychosocial interventions: facilitators and barriers. *Ciencia & Saude Coletiva*, 22(6), pp.1933–1942. Available at: http://search.ebscohost.com/login.aspx?direct=true&db=cmedm&AN=28614513&site=ehost-live. |
| 99 | Temu, F. et al., 2014. Integration of non-communicable diseases in health care: tackling the double burden of disease in African settings. *The Pan African Medical Journal*, 18, p.202. Available at: http://search.ebscohost.com/login.aspx?direct=true&db=cmedm&AN=25419329&site=ehost-live. |
| 100 | Tomori, O., 2015. Will Africa’s future epidemic ride on forgotten lessons from the Ebola epidemic? *BMC Medicine*, 13, p.116. Available at: http://search.ebscohost.com/login.aspx?direct=true&db=cmedm&AN=25976420&site=ehost-live. |
| 101 | Tong, S., 2000. The potential impact of global environmental change on population health. *Australian And New Zealand Journal Of Medicine*, 30(5), pp.618–625. Available at: http://search.ebscohost.com/login.aspx?direct=true&db=cmedm&AN=11108073&site=ehost-live. |
| 102 | Turner, E.L. et al., 2016. A Review of Pediatric Critical Care in Resource-Limited Settings: A Look at Past, Present, and Future Directions. *Frontiers In Pediatrics*, 4, p.5. Available at: http://search.ebscohost.com/login.aspx?direct=true&db=cmedm&AN=26925393&site=ehost-live. |
| 103 | van der Sande, M.A.B., 2003. Cardiovascular disease in sub-Saharan Africa: a disaster waiting to happen. *The Netherlands Journal Of Medicine*, 61(2), pp.32–36. Available at: http://search.ebscohost.com/login.aspx?direct=true&db=cmedm&AN=12735418&site=ehost-live. |
| 104 | Yoo, M., Lee, M. & Tullmann, D., 2016. Perceptions of disaster preparedness among older people in South Korea. *International Journal Of Older People Nursing*, 11(1), pp.18–23. Available at: http://search.ebscohost.com/login.aspx?direct=true&db=cmedm&AN=26179452&site=ehost-live. |
| 105 | Yu, D. et al., 2008. Investment in HIV/AIDS programs: does it help strengthen health systems in developing countries? *Globalization And Health*, 4, p.8. Available at: http://search.ebscohost.com/login.aspx?direct=true&db=cmedm&AN=18796148&site=ehost-live. |
| 106 | Zachariah, R. et al., 2015. Ebola, fragile health systems and tuberculosis care: a call for pre-emptive action and operational research. *The International Journal Of Tuberculosis And Lung Disease: The Official Journal Of The International Union Against Tuberculosis And Lung Disease*, 19(11), pp.1271–1275. Available at: http://search.ebscohost.com/login.aspx?direct=true&db=cmedm&AN=26467577&site=ehost-live. |
| 107 | Zanotti, L., 2010. Cacophonies of aid, failed state building and NGOs in Haiti: setting the stage for disaster, envisioning the future. *Third World Quarterly*, 31(5), pp.755–771. Available at: http://search.ebscohost.com/login.aspx?direct=true&db=cmedm&AN=20821882&site=ehost-live. |
| 108 | Zeng, W. et al., 2017. Assessing the feasibility of introducing health insurance in Afghanistan: a qualitative stakeholder analysis. *BMC Health Services Research*, 17(1), p.157. Available at: http://search.ebscohost.com/login.aspx?direct=true&db=cmedm&AN=28222716&site=ehost-live. |
| 109 | Zhang, J. et al., 2016. Effectiveness and impact of the cross-border healthcare model as implemented by non-governmental organizations: case study of the malaria control programs by health poverty action on the China-Myanmar border. *Infectious Diseases Of Poverty*, 5(1), p.80. Available at: http://search.ebscohost.com/login.aspx?direct=true&db=cmedm&AN=27581190&site=ehost-live. |
| 110 | Zoraster, R.M., 2010. Vulnerable populations: Hurricane Katrina as a case study. *Prehospital And Disaster Medicine*, 25(1), pp.74–78. Available at: http://search.ebscohost.com/login.aspx?direct=true&db=cmedm&AN=20405467&site=ehost-live. |
| 111 | Shroff, Z. et al. (2015). Incorporating research evidence into decision-making processes: researcher and decision-maker perceptions from five low- and middle-income countries. *Health Research Policy and Systems*. 13:70. Available from: https://doi.org/10.1186/s12961-015-0059-y |
| 112 | Colombini, M. et al. (2011). One stop crisis centres: a policy analysis of the Malaysian response to intimate partner violence. *Health Research Policy and Systems*. 9:25. Available from: http://dx.doi.org/10.1186%2F1478-4505-9-25 |
| 113 | Agyepong, I.A. et al. (2017). Spanning maternal, newborn and child health (MNCH) and health systems research boundaries: conducive and limiting health systems factors to improving MNCH outcomes in West Africa. *Health Research Policy and Systems*. 15 (Suppl 1):54. Available from: https://doi.org/10.1186/s12961-017-0212-x |
| 114 | Godt, S. et al. (2017). The change-makers of West Africa. *Health Research Policy and Systems*. 15(Suppl 1): 52. Available from: https://doi.org/10.1186/s12961-017-0208-6 |
| 115 | Olivier de Sardan, J-P. et al. (2017). Travelling models and the challenge of pragmatic contexts and practical norms: the case of maternal health. *Health Research Policy and Systems*. 15(Suppl 1): 60. Available from: https://doi.org/10.1186/s12961-017-0213-9 |
| 116 | Balabanova, D. et al. (2010). What can global health institutions do to help strengthen health systems in low income countries? *Health Research Policy and Systems*. 8:22. Available from: https://doi.org/10.1186/1478-4505-8-22 |
| 117 | Atun, R. et al. (2010). A systematic review of the evidence on integration of targeted health interventions into health systems. *Health Policy and Planning*. 25(1): 1-14. Available from: https://doi.org/10.1093/heapol/czp053 |
| 118 | Jacobs, B. et al. (2012). Addressing access barriers to health services: an analytical framework for selecting appropriate interventions in low-income Asian countries. *Health Policy and Planning*. 27(4): 288-300. Available from: https://doi.org/10.1093/heapol/czr038 |
| 119 | Paul, E. et al. (2014). Aid for health in times of political unrest in Mali: does donors' way of intervening allow protecting people's health? *Health Policy and Planning*. 29(8): 1071-1074. Available from: https://doi.org/10.1093/heapol/czt082 |
| 120 | Ssengooba, F. et al. (2017). Application of social network analysis in the assessment of organization infrastructure for service delivery: a three district case study from post-conflict northern Uganda. *Health Policy and Planning*. 32(8): 1193-1202. Available from: https://doi.org/10.1093/heapol/czx071 |
| 121 | Alonge, O. et al. (2015). Assessing the pro-poor effect of different contracting schemes for health services on health facilities in rural Afghanistan. *Health Policy and Planning*. 30(10): 1229-1242. Available from: https://doi.org/10.1093/heapol/czu127 |
| 122 | Vargas, I. et al. (2016). Barriers to healthcare coordination in market-based and decentralized public health systems: a qualitative study in healthcare networks of Colombia and Brazil. *Health Policy and Planning*. 31(6): 736-748. Available from: https://doi.org/10.1093/heapol/czv126 |
| 123 | Moran, A.C. et al. (2012). Benchmarks to measure readiness to integrate and scale up newborn survival interventions. *Health Policy and Planning*. 27(Suppl 3): iii29-iii39. Available from: https://doi.org/10.1093/heapol/czs046 |
| 124 | Tynan, A. et al. (2014). Building social currency with foreskin cuts: a coping mechanism of Papua New Guinea health workers and the implications for new programmes. *Health Policy and Planning*. 29(7): 902-911. Available from: https://doi.org/10.1093/heapol/czt072 |
| 125 | Svoronos, T. et al. (2015). Can the health system deliver? Determinants of rural Liberians' confidence in health care. *Health Policy and Planning*. 30(7): 823-829. Available from: https://doi.org/10.1093/heapol/czu065 |
| 126 | Phallkey, R.K. et al. (2015). Challenges with the implementation of an Integrated Disease Surveillance and Response (IDSR) system: systematic review of the lessons learned. *Health Policy and Planning.* 30(1): 131-143. Available from: https://doi.org/10.1093/heapol/czt097 |
| 127 | Molyneux, S. et al. (2012). Community accountability at peripheral health facilities: a review of the empirical literature and development of a conceptual framework. *Health Policy and Planning*. 27(7): 541-554. Available from: https://doi.org/10.1093/heapol/czr083 |
| 128 | Miyake, S. et al. (2017). Community midwifery initiatives in fragile and conflict-affected countries: a scoping review of approaches from recruitment to retention. *Health Policy and Planning*. 32(1): 21-33. Available from: https://doi.org/10.1093/heapol/czw093 |
| 129 | Ashar Malik, M. (2017). Did contracting effect the use of primary health care units in Pakistan? *Health Policy and Planning*. 32(7): 1032-1041. Available from: https://doi.org/10.1093/heapol/czx040 |
| 130 | Takian, A. et al. (2011). Expediency and coincidence in re-engineering a health system: an interpretive approach to formation of family medicine in Iran. *Health Policy and Planning*. 26(2): 163-173. Available from: https://doi.org/10.1093/heapol/czq036 |
| 131 | Fernandes, F.S.L., Raizer, M.V. & Brêtas, A.C.P., 2007. Old, poor and out on the streets: on the road to exclusion. *Revista Latino-Americana De Enfermagem*, 15 Spec No, pp.755–761. Available at: http://search.ebscohost.com/login.aspx?direct=true&db=cmedm&AN=17934581&site=ehost-live. |
| 132 | Ferreira, F.R. et al., 2015. Prevention of birth defects in the pre-conception period: knowledge and practice of health care professionals (nurses and doctors) in a city of Southern Brazil. *Iranian Journal Of Reproductive Medicine*, 13(10), pp.657–664. Available at: http://search.ebscohost.com/login.aspx?direct=true&db=cmedm&AN=26644794&site=ehost-live. |
| 133 | Fotokian, Z. et al., 2017. The empowerment of elderly patients with chronic obstructive pulmonary disease: Managing life with the disease. *Plos One*, 12(4), pp.e0174028–e0174028. Available at: http://search.ebscohost.com/login.aspx?direct=true&db=cmedm&AN=28369069&site=ehost-live. |
| 134 | Francis, L.P., 2010. The physician-patient relationship and a National Health Information network. *The Journal Of Law, Medicine & Ethics: A Journal Of The American Society Of Law, Medicine & Ethics*, 38(1), pp.36–49. Available at: http://search.ebscohost.com/login.aspx?direct=true&db=cmedm&AN=20446982&site=ehost-live. |
| 135 | Fosse, A. et al. (2014). End-of-life expectations and experiences among nursing home patients and their relatives—A synthesis of qualitative studies. *Patient Education and Counseling*. 97(1): 3-9. Available from: https://doi.org/10.1016/j.pec.2014.05.025 |
| 136 | Frogner, B.K., Frech 3rd, H.E. & Parente, S.T., 2015. Comparing efficiency of health systems across industrialized countries: a panel analysis. *BMC Health Services Research*, 15, p.415. Available at: http://search.ebscohost.com/login.aspx?direct=true&db=cmedm&AN=26407626&site=ehost-live. |
| 137 | Frost, A. et al., 2016. An assessment of the barriers to accessing the Basic Package of Health Services (BPHS) in Afghanistan: was the BPHS a success? *Globalization And Health*, 12(1), p.71. Available at: http://search.ebscohost.com/login.aspx?direct=true&db=cmedm&AN=27846910&site=ehost-live. |
| 138 | Fujita, N. et al. (2013). Addressing the human resources crisis: a case study of Cambodia’s efforts to reduce maternal mortality (1980–2012). *BMJ Open*. 3(5): e002685. Available from: https://dx.doi.org/10.1136%2Fbmjopen-2013-002685 |
| 139 | Gammouh, O.S. et al., 2015. Chronic diseases, lack of medications, and depression among Syrian refugees in Jordan, 2013-2014. *Preventing Chronic Disease*, 12, pp.E10–E10. Available at: http://search.ebscohost.com/login.aspx?direct=true&db=cmedm&AN=25633485&site=ehost-live. |
| 140 | Garnelo, L., Sousa, A.B.L. & Silva, C. de O. da, 2017. Health regionalization in Amazonas: progress and challenges. *Ciencia & Saude Coletiva*, 22(4), pp.1225–1234. Available at: http://search.ebscohost.com/login.aspx?direct=true&db=cmedm&AN=28444047&site=ehost-live. |
| 141 | Ghattas, H. et al., 2014. Food insecurity among Iraqi refugees living in Lebanon, 10 years after the invasion of Iraq: data from a household survey. *The British Journal Of Nutrition*, 112(1), pp.70–79. Available at: http://search.ebscohost.com/login.aspx?direct=true&db=cmedm&AN=24739803&site=ehost-live. |
| 142 | Giannoni, M., Franzini, L. & Masiero, G., 2016. Migrant integration policies and health inequalities in Europe. *BMC Public Health*, 16, p.463. Available at: http://search.ebscohost.com/login.aspx?direct=true&db=cmedm&AN=27250252&site=ehost-live. |
| 143 | Ginsburg, O. et al., 2017. The global burden of women’s cancers: a grand challenge in global health. *Lancet (London, England)*, 389(10071), pp.847–860. Available at: http://search.ebscohost.com/login.aspx?direct=true&db=cmedm&AN=27814965&site=ehost-live. |
| 144 | Goodhand, J., 2010. Stabilising a victor’s peace? Humanitarian action and reconstruction in eastern Sri Lanka. *Disasters*, 34 Suppl 3, pp.S342–S367. Available at: http://search.ebscohost.com/login.aspx?direct=true&db=cmedm&AN=20846349&site=ehost-live. |
| 145 | Goosby, E. et al., 2012. Raising the bar: PEPFAR and new paradigms for global health. *Journal Of Acquired Immune Deficiency Syndromes (1999)*, 60 Suppl 3, pp.S158–S162. Available at: http://search.ebscohost.com/login.aspx?direct=true&db=cmedm&AN=22797738&site=ehost-live. |
| 146 | Gopalan, S.S., Das, A. & Howard, N., 2017. Maternal and neonatal service usage and determinants in fragile and conflict-affected situations: a systematic review of Asia and the Middle-East. *BMC Women’s Health*, 17(1), p.20. Available at: http://search.ebscohost.com/login.aspx?direct=true&db=cmedm&AN=28298198&site=ehost-live. |
| 147 | Gostin, L.O. & Friedman, E.A., 2015. A retrospective and prospective analysis of the west African Ebola virus disease epidemic: robust national health systems at the foundation and an empowered WHO at the apex. *Lancet (London, England)*, 385(9980), pp.1902–1909. Available at: http://search.ebscohost.com/login.aspx?direct=true&db=cmedm&AN=25987158&site=ehost-live. |
| 148 | Graves, C.M., Haakenstad, A. & Dieleman, J.L., 2015. Tracking development assistance for health to fragile states: 2005-2011. *Globalization And Health*, 11, p.12. Available at: http://search.ebscohost.com/login.aspx?direct=true&db=cmedm&AN=25886046&site=ehost-live. |
| 149 | Griffiths, K. et al., 2013. Public health responses to a dengue outbreak in a fragile state: a case study of Nepal. *Journal Of Tropical Medicine*, 2013, p.158462. Available at: http://search.ebscohost.com/login.aspx?direct=true&db=cmedm&AN=23690789&site=ehost-live. |
| 150 | Guha-Sapir, D. & van Panhuis, W.G., 2003. The importance of conflict-related mortality in civilian populations. *Lancet (London, England)*, 361(9375), pp.2126–2128. Available at: http://search.ebscohost.com/login.aspx?direct=true&db=cmedm&AN=12826439&site=ehost-live. |
| 151 | Haar, R.J. & Rubenstein, L.S. (2012). Health in fragile and post-conflict states: a review of current understanding and challenges ahead. *Medicine, Conflict and Survival*. 28(4): 289-316. Available from: https://doi.org/10.1080/13623699.2012.743311 |
| 152 | Harris, B. et al., 2014. Adverse or acceptable: negotiating access to a post-apartheid health care contract. *Globalization And Health*, 10, p.35. Available at: http://search.ebscohost.com/login.aspx?direct=true&db=cmedm&AN=24885882&site=ehost-live. |
| 153 | Heggen, K. & Wellard, S., 2004. Increased unintended patient harm in nursing practise as a consequence of the dominance of economic discourses. *International Journal Of Nursing Studies*, 41(3), pp.293–298. Available at: http://search.ebscohost.com/login.aspx?direct=true&db=cmedm&AN=14967186&site=ehost-live. |
| 154 | Helou, M. & Rizk, G.A., 2016. State of family medicine practice in Lebanon. *Journal Of Family Medicine And Primary Care*, 5(1), pp.51–55. Available at: http://search.ebscohost.com/login.aspx?direct=true&db=cmedm&AN=27453843&site=ehost-live. |
| 155 | Hennink, M. & McFarland, D.A., 2013. A delicate web: household changes in health behaviour enabled by microcredit in Burkina Faso. *Global Public Health*, 8(2), pp.144–158. Available at: http://search.ebscohost.com/login.aspx?direct=true&db=cmedm&AN=23327537&site=ehost-live. |
| 156 | Heritage, Z. & Green, G., 2013. European national healthy city networks: the impact of an elite epistemic community. *Journal Of Urban Health: Bulletin Of The New York Academy Of Medicine*, 90 Suppl 1, pp.154–166. Available at: http://search.ebscohost.com/login.aspx?direct=true&db=cmedm&AN=23283684&site=ehost-live. |
| 157 | Hessini, L., Brookman-Amissah, E. & Crane, B.B., 2006. Global policy change and women’s access to safe abortion: the impact of the World Health Organization’s guidance in Africa. *African Journal Of Reproductive Health*, 10(3), pp.14–27. Available at: http://search.ebscohost.com/login.aspx?direct=true&db=cmedm&AN=17518128&site=ehost-live. |
| 158 | Hetzel, M.W. et al., 2008. Malaria risk and access to prevention and treatment in the paddies of the Kilombero Valley, Tanzania. *Malaria Journal*, 7, p.7. Available at: http://search.ebscohost.com/login.aspx?direct=true&db=cmedm&AN=18184430&site=ehost-live. |
| 159 | Hodges, M.E. et al., 2011. Neglected tropical disease control in post-war Sierra Leone using the Onchocerciasis Control Programme as a platform. *International Health*, 3(2), pp.69–74. Available at: http://search.ebscohost.com/login.aspx?direct=true&db=cmedm&AN=24038179&site=ehost-live. |
| 160 | Husain, T. & Chaudhary, J.R., 2008. Human health risk assessment due to global warming--a case study of the Gulf countries. *International Journal Of Environmental Research And Public Health*, 5(4), pp.204–212. Available at: http://search.ebscohost.com/login.aspx?direct=true&db=cmedm&AN=19190352&site=ehost-live. |
| 161 | Hussain, Z. & Sullivan, R., 2017. Tobacco in post-conflict settings: the case of Iraq. *Ecancermedicalscience*, 11, p.735. Available at: http://search.ebscohost.com/login.aspx?direct=true&db=cmedm&AN=28596801&site=ehost-live. |
| 162 | Irons, T.G. & Moore, K.S., 2015. The importance of health insurance and the safety net in rural communities. *North Carolina Medical Journal*, 76(1), pp.50–53. Available at: http://search.ebscohost.com/login.aspx?direct=true&db=cmedm&AN=25621483&site=ehost-live. |
| 163 | Lee, K., Solts, B. & Burns, J., 2002. Investigating the psychosocial impact of anti-HIV combination therapies. *AIDS Care*, 14(6), pp.851–857. Available at: http://search.ebscohost.com/login.aspx?direct=true&db=cmedm&AN=12511217&site=ehost-live. |
| 164 | Lewis, J.M. et al., 2014. Social capital in a lower socioeconomic palliative care population: a qualitative investigation of individual, community and civic networks and relations. *BMC Palliative Care*, 13, p.30. Available at: http://search.ebscohost.com/login.aspx?direct=true&db=cmedm&AN=24959101&site=ehost-live. |
| 165 | Liang, H. & Xue, Y., 2004. Investigating public health emergency response information system initiatives in China. *International Journal Of Medical Informatics*, 73(9–10), pp.675–685. Available at: http://search.ebscohost.com/login.aspx?direct=true&db=cmedm&AN=15325324&site=ehost-live. |
| 166 | Liu, H., Behr, J.G. & Diaz, R., 2016. Population vulnerability to storm surge flooding in coastal Virginia, USA. *Integrated Environmental Assessment And Management*, 12(3), pp.500–509. Available at: http://search.ebscohost.com/login.aspx?direct=true&db=cmedm&AN=26295749&site=ehost-live. |
| 167 | Loretti, A. & Tegegn, Y., 1996. Disasters in Africa: old and new hazards and growing vulnerability. *World Health Statistics Quarterly. Rapport Trimestriel De Statistiques Sanitaires Mondiales*, 49(3–4), pp.179–184. Available at: http://search.ebscohost.com/login.aspx?direct=true&db=cmedm&AN=9170231&site=ehost-live. |
| 168 | Lunardi, V.L., 2013. Moral distress: an innovative and important subject to study in Brazil : commentary on “A reflection on moral distress in nursing together with a current application of the concept” by Andrew Jameton. *Journal Of Bioethical Inquiry*, 10(3), pp.309–312. Available at: http://search.ebscohost.com/login.aspx?direct=true&db=cmedm&AN=23877380&site=ehost-live. |
| 169 | Maciocco, G. & Stefanini, A., 2007. From Alma-Ata to the Global Fund: the history of international health policy TT - De Alma-Ata ao Fundo Global: a história da política internacional de saúde. *Rev. bras. saúde matern. infant*, 7(4), pp.479–486. Available at: http://www.scielo.br/scielo.php?script=sci_arttext&pid=S1519-38292007000400016. |
| 170 | Mackersie, R.C., 2006. Field triage, and the fragile supply of “optimal resources” for the care of the injured patient. *Prehospital Emergency Care: Official Journal Of The National Association Of EMS Physicians And The National Association Of State EMS Directors*, 10(3), pp.347–350. Available at: http://search.ebscohost.com/login.aspx?direct=true&db=cmedm&AN=16801277&site=ehost-live. |
| 171 | Mackey, T.K. & Strathdee, S.A., 2015. Responding to the public health consequences of the Ukraine crisis: an opportunity for global health diplomacy. *Journal Of The International AIDS Society*, 18, p.19410. Available at: http://search.ebscohost.com/login.aspx?direct=true&db=cmedm&AN=25787347&site=ehost-live. |
| 172 | Manderson, L., Block, E. & Mkhwanazi, N., 2016. Fragility, fluidity, and resilience: caregiving configurations three decades into AIDS. *AIDS Care*, 28 Suppl 4, pp.1–7. Available at: http://search.ebscohost.com/login.aspx?direct=true&db=cmedm&AN=27410678&site=ehost-live. |
| 173 | Marcelin, L.H., Cela, T. & Shultz, J.M., 2016. Haiti and the politics of governance and community responses to Hurricane Matthew. *Disaster Health*, 3(4), pp.151–161. Available at: http://search.ebscohost.com/login.aspx?direct=true&db=cmedm&AN=28321361&site=ehost-live. |
| 174 | Mark, A. & Jones, M., 2013. Thinking through health capacity development for Fragile States. *The International Journal Of Health Planning And Management*, 28(3), pp.269–289. Available at: http://search.ebscohost.com/login.aspx?direct=true&db=cmedm&AN=23047746&site=ehost-live. |
| 175 | Martin, G.P. & Finn, R., 2011. Patients as team members: opportunities, challenges and paradoxes of including patients in multi-professional healthcare teams. *Sociology Of Health & Illness*, 33(7), pp.1050–1065. Available at: http://search.ebscohost.com/login.aspx?direct=true&db=cmedm&AN=21668454&site=ehost-live. |
| 176 | Martinez, W. et al., 2017. Barriers to Integrating Mental Health Services in Community-Based Primary Care Settings in Mexico City: A Qualitative Analysis. *Psychiatric Services (Washington, D.C.)*, 68(5), pp.497–502. Available at: http://search.ebscohost.com/login.aspx?direct=true&db=cmedm&AN=27974004&site=ehost-live. |
| 177 | Masquillier, C. et al., 2015. On the road to HIV/AIDS competence in the household: building a health-enabling environment for people living with HIV/AIDS. *International Journal Of Environmental Research And Public Health*, 12(3), pp.3264–3292. Available at: http://search.ebscohost.com/login.aspx?direct=true&db=cmedm&AN=25794189&site=ehost-live. |
| 178 | Mauch, V. et al., 2010. Structure and management of tuberculosis control programs in fragile states--Afghanistan, DR Congo, Haiti, Somalia. *Health Policy (Amsterdam, Netherlands)*, 96(2), pp.118–127. Available at: http://search.ebscohost.com/login.aspx?direct=true&db=cmedm&AN=20170977&site=ehost-live. |
| 179 | McLellan, A.T. & Meyers, K., 2004. Contemporary addiction treatment: a review of systems problems for adults and adolescents. *Biological Psychiatry*, 56(10), pp.764–770. Available at: http://search.ebscohost.com/login.aspx?direct=true&db=cmedm&AN=15556121&site=ehost-live. |
| 180 | Mecha, J.O. et al., 2016. Trends in clinical characteristics and outcomes of Pre-ART care at a large HIV clinic in Nairobi, Kenya: a retrospective cohort study. *AIDS Research And Therapy*, 13, p.38. Available at: http://search.ebscohost.com/login.aspx?direct=true&db=cmedm&AN=27895697&site=ehost-live. |
| 181 | Melgaard, B. et al., 1998. Disease eradication and health systems development. *Bulletin Of The World Health Organization*, 76 Suppl 2, pp.26–31. Available at: http://search.ebscohost.com/login.aspx?direct=true&db=cmedm&AN=10063670&site=ehost-live. |
| 182 | Milstien, J. & Lambert, S., 2002. Emergency response vaccines--a challenge for the public sector and the vaccine industry. *Vaccine*, 21(1–2), pp.146–154. Available at: http://search.ebscohost.com/login.aspx?direct=true&db=cmedm&AN=12443673&site=ehost-live. |
| 183 | Miyoshi, M. et al., 2005. Nutritional status of children in rural Lao PDR: who are the most vulnerable? *European Journal Of Clinical Nutrition*, 59(7), pp.887–890. Available at: http://search.ebscohost.com/login.aspx?direct=true&db=cmedm&AN=15915154&site=ehost-live. |
| 184 | Mobula, M.L. et al., 2015. Need for Reinforced Strategies to Support Delivery of HIV Clinical Services During the Ebola Outbreak in Guinea, Liberia, and Sierra Leone. *Disaster Medicine And Public Health Preparedness*, 9(5), pp.522–526. Available at: http://search.ebscohost.com/login.aspx?direct=true&db=cmedm&AN=25782527&site=ehost-live. |
| 185 | Moos, R.H., 2003. Social contexts: transcending their power and their fragility. *American Journal Of Community Psychology*, 31(1–2), pp.1–13. Available at: http://search.ebscohost.com/login.aspx?direct=true&db=cmedm&AN=12741686&site=ehost-live. |
| 186 | Morrison, J.B. & Rudolph, J.W., 2011. Learning from accident and error: avoiding the hazards of workload, stress, and routine interruptions in the emergency department. *Academic Emergency Medicine: Official Journal Of The Society For Academic Emergency Medicine*, 18(12), pp.1246–1254. Available at: http://search.ebscohost.com/login.aspx?direct=true&db=cmedm&AN=22168187&site=ehost-live. |
| 187 | Morse, A., 2002. Bioterrorism preparedness for local health departments. *Journal Of Community Health Nursing*, 19(4), pp.203–211. Available at: http://search.ebscohost.com/login.aspx?direct=true&db=cmedm&AN=12494741&site=ehost-live. |
| 188 | Muggah, R., 2010. The effects of stabilisation on humanitarian action in Haiti. *Disasters*, 34 Suppl 3, pp.S444–S463. Available at: http://search.ebscohost.com/login.aspx?direct=true&db=cmedm&AN=20846354&site=ehost-live. |
| 189 | Mutale, W. et al., 2013. Systems thinking in practice: the current status of the six WHO building blocks for health system strengthening in three BHOMA intervention districts of Zambia: a baseline qualitative study. *BMC Health Services Research*, 13, p.291. Available at: http://search.ebscohost.com/login.aspx?direct=true&db=cmedm&AN=23902601&site=ehost-live. |
| 190 | Negin, J. & Martiniuk, A., 2012. Sector wide approaches for health in small island states: lessons learned from the Solomon Islands. *Global Public Health*, 7(2), pp.137–148. Available at: http://search.ebscohost.com/login.aspx?direct=true&db=cmedm&AN=21736517&site=ehost-live. |
| 191 | Newbrander, W. et al., 2012. A tool for assessing management capacity at the decentralized level in a fragile state. *The International Journal Of Health Planning And Management*, 27(4), pp.276–294. Available at: http://search.ebscohost.com/login.aspx?direct=true&db=cmedm&AN=22034286&site=ehost-live. |
| 192 | WHO. (2015). *2015 WHO Strategic Response Plan: West Africa Ebola Outbreak*. World Health Organization. Available from: http://www.who.int/csr/resources/publications/ebola/ebola-strategic-plan/en/ [Accessed 8th November 2018] |
| 193 | World Health Organization & UNICEF/UNDP/World Bank/WHO Special Programme for Research and Training in Tropical Diseases. (‎2012)‎. *Assessment of research needs for public health adaptation to social, environmental and climate change impacts on vector-borne diseases in Africa: an informal expert consultation convened by the Special Programme for Research and Training in Tropical Diseases (‎TDR)‎, Addis Ababa, Ethiopia, February 27-29, 2012*. World Health Organization. Available from: http://www.who.int/iris/handle/10665/76282 [Accessed 8th November 2018] |
| 194 | Elyan, D.S. et al. (2014). Capacity building of public health laboratories in Afghanistan: challenges and successes (2007–2011). *Eastern Mediterranean Health Journal*. 20(2): 112-119. Available from: https://doi.org/10.26719/2014.20.2.112 |
| 195 | WHO. (2015). *Ebola Virus Disease Preparedness: Taking Stock and Moving Forward*. World Health Organization. Available from: http://www.who.int/csr/resources/publications/ebola/preparedness-meeting-report/en/ [Accessed 8th November 2018] |
| 196 | Hanna, R. & Oliva, P. (2016). Implications of Climate Change for Children in Developing Countries. The Future of Children 26(1), 115-132. Available from: https://doi.org/10.1353/foc.2016.0006 |
| 197 | Taverne, B. (2015). Preparing for Ebola outbreaks: not without the social sciences! *Global Health Promotion*. 22(2): 5-6. Available from: https://doi.org/10.1177/2F1757975915582298 |
| 198 | Keys, H. et al. (2015). Liberia—Moving Beyond “Ebola Free”. *Emerging Infectious Diseases*. 21(11): 2091-2092. Available from: http://dx.doi.org/10.3201%2Feid2111.151322 |
| 199 | Mwaura, P. (2008). Turmoil disrupts AIDS care in Kenya. *Bulletin of the World Health Organization*. 86(3): 168-169. Available from: http://www.who.int/bulletin/volumes/86/3/08-030308.pdf [Accessed 8th November 2018] |
| 200 | Cooper, J.L. (2015). Mental health and psychosocial support in the face of Ebola in Liberia: the personal and professional intersect. A personal account. *Intervention*. 13(1): 45-84. Available from: https://www.interventionjournal.com/sites/default/files/Mental_health_and_psychosocial_support_in_the_face.7.pdf [Accessed 8th November 2018] |
| 201 | Newbrander, W., Waldman, R. & Shepherd-Banigan, M., 2011. Rebuilding and strengthening health systems and providing basic health services in fragile states. *Disasters*, 35(4), pp.639–660. Available at: http://search.ebscohost.com/login.aspx?direct=true&db=cmedm&AN=21913929&site=ehost-live. |
| 202 | Newman, R.D., 2012. Relegating malaria resurgences to history. *Malaria Journal*, 11, p.123. Available at: http://search.ebscohost.com/login.aspx?direct=true&db=cmedm&AN=22531295&site=ehost-live. |
| 203 | Nielsen, J., Prudhon, C. & de Radigues, X., 2011. Trends in malnutrition and mortality in Darfur, Sudan, between 2004 and 2008: a meta-analysis of publicly available surveys. *International Journal Of Epidemiology*, 40(4), pp.971–984. Available at: http://search.ebscohost.com/login.aspx?direct=true&db=cmedm&AN=21296853&site=ehost-live. |
| 204 | Noé, A. et al., 2017. Knowledge, attitudes and practices regarding tuberculosis care among health workers in Southern Mozambique. *BMC Pulmonary Medicine*, 17(1), p.2. Available at: http://search.ebscohost.com/login.aspx?direct=true&db=cmedm&AN=28056943&site=ehost-live. |
| 205 | Ntsekhe, M. & Hakim, J., 2005. Impact of human immunodeficiency virus infection on cardiovascular disease in Africa. *Circulation*, 112(23), pp.3602–3607. Available at: http://search.ebscohost.com/login.aspx?direct=true&db=cmedm&AN=16330702&site=ehost-live. |
| 206 | O’Connell, B., Bailey, S. & Walker, A., 2003. Promoting the health and well being of older carers: a proactive strategy. *Australian Health Review: A Publication Of The Australian Hospital Association*, 26(2), pp.78–86. Available at: http://search.ebscohost.com/login.aspx?direct=true&db=cmedm&AN=15368839&site=ehost-live. |
| 207 | O’Dempsey, T. & Munslow, B., 2009. “Mind the gap!” rethinking the role of health in the emergency and development divide. *The International Journal Of Health Planning And Management*, 24 Suppl 1, pp.S21–S29. Available at: http://search.ebscohost.com/login.aspx?direct=true&db=cmedm&AN=19957308&site=ehost-live. |
| 208 | Oliveira, C. & Russo, G., 2015. Vertical interventions and system effects; have we learned anything from past experiences? *The Pan African Medical Journal*, 21, p.262. Available at: http://search.ebscohost.com/login.aspx?direct=true&db=cmedm&AN=26523197&site=ehost-live. |
| 209 | Omenka, C. & Zarowsky, C., 2013. “No one knows what will happen after these five years”: narratives of ART, access and agency in Nigeria. *Global Health Promotion*, 20(1 Suppl), pp.45–50. Available at: http://search.ebscohost.com/login.aspx?direct=true&db=cmedm&AN=23549702&site=ehost-live. |
| 210 | Omoleke, S.A., Mohammed, I. & Saidu, Y., 2016. Ebola Viral Disease in West Africa: A Threat to Global Health, Economy and Political Stability. *Journal Of Public Health In Africa*, 7(1), p.534. Available at: http://search.ebscohost.com/login.aspx?direct=true&db=cmedm&AN=28299152&site=ehost-live. |
| 211 | Orya, E. et al., 2017. Strengthening close to community provision of maternal health services in fragile settings: an exploration of the changing roles of TBAs in Sierra Leone and Somaliland. *BMC Health Services Research*, 17(1), p.460. Available at: http://search.ebscohost.com/login.aspx?direct=true&db=cmedm&AN=28679383&site=ehost-live. |
| 212 | Paintain, L.S. et al., 2013. Sustaining fragile gains: the need to maintain coverage with long-lasting insecticidal nets for malaria control and likely implications of not doing so. *Plos One*, 8(12), pp.e83816–e83816. Available at: http://search.ebscohost.com/login.aspx?direct=true&db=cmedm&AN=24386283&site=ehost-live. |
| 213 | Palmer, D., 2006. Tackling Malawi’s human resources crisis. *Reproductive Health Matters*, 14(27), pp.27–39. Available at: http://search.ebscohost.com/login.aspx?direct=true&db=cmedm&AN=16713877&site=ehost-live. |
| 214 | Pang, T. & Peeling, R.W., 2007. Diagnostic tests for infectious diseases in the developing world: two sides of the coin. *Transactions Of The Royal Society Of Tropical Medicine And Hygiene*, 101(9), pp.856–857. Available at: http://search.ebscohost.com/login.aspx?direct=true&db=cmedm&AN=17544047&site=ehost-live. |
| 215 | Peckham, S. et al., 2014. Commissioning for long-term conditions: hearing the voice of and engaging users – a qualitative multiple case study. Available at: http://search.ebscohost.com/login.aspx?direct=true&db=cmedm&AN=25642540&site=ehost-live. |
| 216 | Peltzer, J.N., Domian, E.W. & Teel, C.S., 2016. Infected Lives: Lived Experiences of Young African American HIV-Positive Women. *Western Journal Of Nursing Research*, 38(2), pp.216–230. Available at: http://search.ebscohost.com/login.aspx?direct=true&db=cmedm&AN=25239137&site=ehost-live. |
| 217 | Pereira, S.M. et al., 2012. Improving access to eye health services in rural Timor-Leste. *Rural And Remote Health*, 12, p.2095. Available at: http://search.ebscohost.com/login.aspx?direct=true&db=cmedm&AN=22994876&site=ehost-live. |
| 218 | Phillips, A.F. & Pirkle, C.M., 2011. Moving beyond behaviour: advancing HIV risk prevention epistemologies and interventions (A report on the state of the literature). *Global Public Health*, 6(6), pp.577–592. Available at: http://search.ebscohost.com/login.aspx?direct=true&db=cmedm&AN=21790501&site=ehost-live. |
| 219 | Phillips, C.B. & Benson, J., 2007. Better primary health care for refugees - catch up immunisation. *Australian Family Physician*, 36(6), p.440. Available at: http://search.ebscohost.com/login.aspx?direct=true&db=cmedm&AN=17565403&site=ehost-live. |
| 220 | Pingali, P., Alinovi, L. & Sutton, J., 2005. Food security in complex emergencies: enhancing food system resilience. *Disasters*, 29 Suppl 1, pp.S5–S24. Available at: http://search.ebscohost.com/login.aspx?direct=true&db=cmedm&AN=15910676&site=ehost-live. |
| 221 | Pozza dos Santos, B. et al., 2016. The bond as a soft technology in the daily routine of the Family Health Strategy: perception of the user TT - El vínculo como tecnología leve en el cotidiano de la Estrategia de Salud de la Familia: el mirar del usuario TT - O vínculo como tecnologia le. *Invest Educ Enferm*, 34(1), pp.189–197. Available at: http://www.scielo.org.co/scielo.php?script=sci_arttext&pid=S0120-53072016000100021. |
| 222 | Rabbani, F. et al., 2016. Schools of public health in low and middle-income countries: an imperative investment for improving the health of populations? *BMC Public Health*, 16, p.941. Available at: http://search.ebscohost.com/login.aspx?direct=true&db=cmedm&AN=27604901&site=ehost-live. |
| 223 | Rabkin, M., El-Sadr, W.M. & De Cock, K.M., 2009. The impact of HIV scale-up on health systems: A priority research agenda. *Journal Of Acquired Immune Deficiency Syndromes (1999)*, 52 Suppl 1, pp.S6–S11. Available at: http://search.ebscohost.com/login.aspx?direct=true&db=cmedm&AN=19858943&site=ehost-live. |
| 224 | Regmi, K., Gilbert, R. & Thunhurst, C., 2015. How can health systems be strengthened to control and prevent an Ebola outbreak? A narrative review. *Infection Ecology & Epidemiology*, 5, p.28877. Available at: http://search.ebscohost.com/login.aspx?direct=true&db=cmedm&AN=26609690&site=ehost-live. |
| 225 | Richards, E. et al., 2013. Neglected older women and men: Exploring age and gender as structural drivers of HIV among people aged over 60 in Uganda. *African Journal Of AIDS Research: AJAR*, 12(2), pp.71–78. Available at: http://search.ebscohost.com/login.aspx?direct=true&db=cmedm&AN=25871376&site=ehost-live. |
| 226 | Ricketts, T.C., 2000. The changing nature of rural health care. *Annual Review Of Public Health*, 21, pp.639–657. Available at: http://search.ebscohost.com/login.aspx?direct=true&db=cmedm&AN=10884968&site=ehost-live. |
| 227 | Rigotto, R.M., 2009. Exploring fragility: industrial delocalization, occupational and environmental risks, and non-governmental organizations. *International Journal Of Environmental Research And Public Health*, 6(3), pp.980–998. Available at: http://search.ebscohost.com/login.aspx?direct=true&db=cmedm&AN=19440428&site=ehost-live. |
| 228 | Rockers, P.C., Kruk, M.E. & Laugesen, M.J., 2012. Perceptions of the health system and public trust in government in low- and middle-income countries: evidence from the World Health Surveys. *Journal Of Health Politics, Policy And Law*, 37(3), pp.405–437. Available at: http://search.ebscohost.com/login.aspx?direct=true&db=cmedm&AN=22323234&site=ehost-live. |
| 229 | Rodewald, L.E. et al., 2006. Vaccine supply problems: a perspective of the Centers for Disease Control and Prevention. *Clinical Infectious Diseases: An Official Publication Of The Infectious Diseases Society Of America*, 42 Suppl 3, pp.S104–S110. Available at: http://search.ebscohost.com/login.aspx?direct=true&db=cmedm&AN=16447130&site=ehost-live. |
| 230 | Rogers, G.D. et al., 2005. Caring for a marginalised community: the costs of engaging with culture and complexity. *The Medical Journal Of Australia*, 183(10 Suppl), pp.S59–S63. Available at: http://search.ebscohost.com/login.aspx?direct=true&db=cmedm&AN=16296954&site=ehost-live. |
| 231 | Rogers, W.A. & Walker, M.J., 2016. Fragility, uncertainty, and healthcare. *Theoretical Medicine And Bioethics*, 37(1), pp.71–83. Available at: http://search.ebscohost.com/login.aspx?direct=true&db=cmedm&AN=26906556&site=ehost-live. |
| 232 | Rosewell, A. et al., 2013. Mobile phone-based syndromic surveillance system, Papua New Guinea. *Emerging Infectious Diseases*, 19(11), pp.1811–1818. Available at: http://search.ebscohost.com/login.aspx?direct=true&db=cmedm&AN=24188144&site=ehost-live. |
| 233 | Rushton, C.H., Reina, M.L. & Reina, D.S., 2007. Building trustworthy relationships with critically ill patients and families. *AACN Advanced Critical Care*, 18(1), pp.19–30. Available at: http://search.ebscohost.com/login.aspx?direct=true&db=cmedm&AN=17284945&site=ehost-live. |
| 234 | Salama, P. et al., 2014. Post-crisis Zimbabwe’s innovative financing mechanisms in the social sectors: a practical approach to implementing the new deal for engagement in fragile states. *BMC International Health And Human Rights*, 14, p.35. Available at: http://search.ebscohost.com/login.aspx?direct=true&db=cmedm&AN=25494877&site=ehost-live. |
| 235 | Salvage, J., 2007. Casualties of war. *Nursing Standard (Royal College Of Nursing (Great Britain): 1987)*, 21(50), pp.20–22. Available at: http://search.ebscohost.com/login.aspx?direct=true&db=cmedm&AN=17896571&site=ehost-live. |
| 236 | Samb, B. et al. (2009). An assessment of interactions between global health initiatives and country health systems. *The Lancet. 373(9681): 2137-2169.* Available from: https://doi.org/10.1016/S0140-6736(09)60919-3 |
| 237 | Sawaguchi, A. & Sawaguchi, T., 2002. Japanese national SIDS project: 1998-2000 research for the improvement of infant mortality. *Forensic Science International*, 130 Suppl, pp.S1–S7. Available at: http://search.ebscohost.com/login.aspx?direct=true&db=cmedm&AN=12350295&site=ehost-live. |
| 238 | Schlesinger, M., 2002. On values and democratic policy making: the deceptively fragile consensus around market-oriented medical care. *Journal Of Health Politics, Policy And Law*, 27(6), pp.889–925. Available at: http://search.ebscohost.com/login.aspx?direct=true&db=cmedm&AN=12556021&site=ehost-live. |
| 239 | Sen, K. & Bonita, R., 2000. Global health status: two steps forward, one step back. *Lancet (London, England)*, 356(9229), pp.577–582. Available at: http://search.ebscohost.com/login.aspx?direct=true&db=cmedm&AN=10950247&site=ehost-live. |
| 240 | Shippy, R.A. & Karpiak, S.E., 2005. The aging HIV/AIDS population: fragile social networks. *Aging & Mental Health*, 9(3), pp.246–254. Available at: http://search.ebscohost.com/login.aspx?direct=true&db=cmedm&AN=16019278&site=ehost-live. |
| 241 | Silva, V.N., D’Oliveira, A.F. & Mesquita, F., 2007. [Vulnerability to HIV among female injecting drug users]. *Revista De Saude Publica*, 41 Suppl 2, pp.22–30. Available at: http://search.ebscohost.com/login.aspx?direct=true&db=cmedm&AN=18094783&site=ehost-live. |
| 242 | Curry, D.W. et al. (2015). Delivering High-Quality Family Planning Services in Crisis-Affected Settings II: Results. *Global Health: Science and Practice*. 3(1): 25-33. Available from: https://dx.doi.org/10.9745%2FGHSP-D-14-00112 |
| 243 | Potts, M. (2014). Getting family planning and population back on track. *Global Health: Science and Practice*. 2(2): 145-151. Available from: https://dx.doi.org/10.9745%2FGHSP-D-14-00012 |
| 244 | Steinglass, R. (2013). Routine immunization: an essential but wobbly platform. *Global Health: Science and Practice*. 1(3): 295-301. Available from: https://dx.doi.org/10.9745%2FGHSP-D-13-00122 |
| 245 | Kamp Dusha C. M. et al. (2013). Chaos as a social determinant of child health: Reciprocal associations? *Social Science and Medicine*. 95: 69-76. Available from: https://doi.org/10.1016/j.socscimed.2013.01.038 |
| 246 | Farmer, J. et al. (2003). Dr. John has gone: assessing health professionals’ contribution to remote rural community sustainability in the UK. *Social Science and Medicine*. 57(4): 673-678. Available from: https://doi.org/10.1016/S0277-9536(02)00410-0 |
| 247 | Schmeer, K.K. (2012). Early childhood economic disadvantage and the health of Hispanic children. *Social Science & Medicine*. 75(8): 1523-1530. Available from: https://doi.org/10.1016/j.socscimed.2012.05.031 |
| 248 | Hospers A.P.N. et al. (2007). Health care delivery systems for older adults: How do the Netherlands and Lebanon compare? *Social Science and Medicine*. 65(1): 1979-1985. Available from: https://doi.org/10.1016/j.socscimed.2007.08.033 |
| 249 | Jones, S., 1994. Food security reserve policy in Ethiopia: a case study of experience and implications. *Disasters*, 18(2), pp.140–151. Available at: http://search.ebscohost.com/login.aspx?direct=true&db=cmedm&AN=8076158&site=ehost-live. |
| 250 | Trani J-T. et al. (2010). Poverty, vulnerability, and provision of healthcare in Afghanistan. *Social Science and Medicine*. 70(11): 1745-1755. Available from: https://doi.org/10.1016/j.socscimed.2010.02.007 |
| 251 | Jennings, M. (2015). The precariousness of the franchise state: Voluntary sector health services and international NGOs in Tanzania, 1960s – mid-1980s. *Social Science and Medicine*. 141: 1-8. Available from: https://doi.org/10.1016/j.socscimed.2015.07.018 |
| 252 | Manuela, J.I. (2012). The influence of stress and social support on depressive symptoms in mothers with young children. *Social Science and Medicine*. 75(11): 2013-2020. Available from: https://doi.org/10.1016/j.socscimed.2012.07.034 |
| 253 | Taylor, S.A.J. (2014). Scaling up nutrition in fragile and conflict-affected states: The pivotal role of governance. *Social Science and Medicine*. 126: 119-127. Available from: https://doi.org/10.1016/j.socscimed.2014.12.016 |
| 254 | Jappah, J. V, 2013. The convergence of American and Nigerian religious conservatism in a biopolitical shaping of Nigeria’s HIV/AIDS prevention programmes. *Global Public Health*, 8(3), pp.312–325. Available at: http://search.ebscohost.com/login.aspx?direct=true&db=cmedm&AN=23391163&site=ehost-live. |
| 255 | Jayasinghe, S., 2009. Contracts to devolve health services in fragile states and developing countries: do ethics matter? *Journal Of Medical Ethics*, 35(9), pp.552–557. Available at: http://search.ebscohost.com/login.aspx?direct=true&db=cmedm&AN=19717694&site=ehost-live. |
| 256 | Kalanda, B., Makwiza, I. & Kemp, J., 2007. Framework for monitoring equity in access and health systems issues in antiretroviral therapy Programmes in southern Africa. *Malawi Medical Journal: The Journal Of Medical Association Of Malawi*, 19(1), pp.20–24. Available at: http://search.ebscohost.com/login.aspx?direct=true&db=cmedm&AN=23878627&site=ehost-live. |
| 257 | Karunamoorthi, K., 2014. The counterfeit anti-malarial is a crime against humanity: a systematic review of the scientific evidence. *Malaria Journal*, 13, p.209. Available at: http://search.ebscohost.com/login.aspx?direct=true&db=cmedm&AN=24888370&site=ehost-live. |
| 258 | Khan, M.S., Hutchison, C. & Coker, R.J., 2017. Risk factors that may be driving the emergence of drug resistance in tuberculosis patients treated in Yangon, Myanmar. *Plos One*, 12(6), pp.e0177999–e0177999. Available at: http://search.ebscohost.com/login.aspx?direct=true&db=cmedm&AN=28614357&site=ehost-live. |
| 259 | Khayatzadeh-Mahani, A. & Takian, A., 2014. Family physician program in Iran: considerations for adapting the policy in urban settings. *Archives Of Iranian Medicine*, 17(11), pp.776–778. Available at: http://search.ebscohost.com/login.aspx?direct=true&db=cmedm&AN=25365620&site=ehost-live. |
| 260 | Kietzman, K.G. et al., 2012. A portrait of older californians with disabilities who rely on public services to remain independent. *Home Health Care Services Quarterly*, 31(4), pp.317–336. Available at: http://search.ebscohost.com/login.aspx?direct=true&db=cmedm&AN=23216515&site=ehost-live. |
| 261 | Kock, L. & Prost, A., 2017. Family Planning and the Samburu: A Qualitative Study Exploring the Thoughts of Men on a Population Health and Environment Programme in Rural Kenya. *International Journal Of Environmental Research And Public Health*, 14(5). Available at: http://search.ebscohost.com/login.aspx?direct=true&db=cmedm&AN=28505083&site=ehost-live. |
| 262 | Kumpel, E. et al., 2016. Urban Water Services in Fragile States: An Analysis of Drinking Water Sources and Quality in Port Harcourt, Nigeria, and Monrovia, Liberia. *The American Journal Of Tropical Medicine And Hygiene*, 95(1), pp.229–238. Available at: http://search.ebscohost.com/login.aspx?direct=true&db=cmedm&AN=27114291&site=ehost-live. |
| 263 | Lago, R.R., Peter, E. & Bógus, C.M., 2017. Harm Reduction and Tensions in Trust and Distrust in a Mental Health Service: A Qualitative Approach. *Substance Abuse Treatment, Prevention, And Policy*, 12(1), p.12. Available at: http://search.ebscohost.com/login.aspx?direct=true&db=cmedm&AN=28270218&site=ehost-live. |
| 264 | Larson, G.S. et al., 2017. Conventional Wisdom versus Actual Outcomes: Challenges in the Conduct of an Ebola Vaccine Trial in Liberia during the International Public Health Emergency. *The American Journal Of Tropical Medicine And Hygiene*, 97(1), pp.10–15. Available at: http://search.ebscohost.com/login.aspx?direct=true&db=cmedm&AN=28719299&site=ehost-live. |
| 265 | Roome, E. et al. (2014). Human resource management in post-conflict health systems: review of research and knowledge gaps. *Conflict and Health*. 8:18. Available from: https://doi.org/10.1186/1752-1505-8-18 |
| 266 | Lassi, Z.S. et al. (2015). Impact of service provision platforms on maternal and newborn health in conflict areas and their acceptability in Pakistan: a systematic review. *Conflict and Health*. 9:25. Available from: https://doi.org/10.1186/s13031-015-0054-5 |
| 267 | Seddiq, K. (2014). Implementing a successful tuberculosis programme within primary care services in a conflict area using the stop TB strategy: Afghanistan case study. *Conflict and Health*. 8:3 Available from: https://doi.org/10.1186/1752-1505-8-3 |
| 268 | Anwari, Z. et al., 2015. Implementing people-centred health systems governance in 3 provinces and 11 districts of Afghanistan: a case study. *Conflict And Health*, 9, p.2. Available at: http://search.ebscohost.com/login.aspx?direct=true&db=cmedm&AN=25904978&site=ehost-live. |
| 269 | Luitel, N.P. et al. (2015). Mental health care in Nepal: current situation and challenges for development of a district mental health care plan. *Conflict and Health*. 9:3. Available from: https://doi.org/10.1186/s13031-014-0030-5 |
| 270 | Aebischer Perone, S. et al. (2017). Non-communicable diseases in humanitarian settings: ten essential questions. *Conflict and Health*. 11:17. Available from: https://doi.org/10.1186/s13031-017-0119-8 |
| 271 | Chu, K. et al. (2010). Surgical care for the direct and indirect victims of violence in the eastern Democratic Republic of Congo. *Conflict and Health*. 4:6 Available from: https://doi.org/10.1186/1752-1505-4-6 |
| 272 | Hill, P.S. et al. (2014). The “empty void” is a crowded space: health service provision at the margins of fragile and conflict affected states. *Conflict and Health*. 8:20. Available from: https://doi.org/10.1186/1752-1505-8-20 |
| 273 | Salama, P. & Alwan, A. (2016). Building health systems in fragile states: the instructive example of Afghanistan. *The Lancet Global Health*. 4(6): E351-E352. Available from: https://doi.org/10.1016/S2214-109X(16)30067-5 |
| 274 | Fujita, N. et al., 2013. Addressing the human resources crisis: a case study of Cambodia’s efforts to reduce maternal mortality (1980-2012). *BMJ Open*, 3(5). Available at: http://search.ebscohost.com/login.aspx?direct=true&db=cmedm&AN=23674446&site=ehost-live. |
| 275 | Risso-Gill, I. et al. (2013). Health system strengthening in Myanmar during political reforms: perspectives from international agencies. *Health Policy and Planning*. 29(4): 466-474. Available from: https://doi.org/10.1093/heapol/czt037 |
| 276 | Casas, L., 2009. Invoking conscientious objection in reproductive health care: evolving issues in Peru, Mexico and Chile. *Reproductive Health Matters*, 17(34), pp.78–87. Available at: http://search.ebscohost.com/login.aspx?direct=true&db=cmedm&AN=19962641&site=ehost-live. |
| 277 | Cavalcanti, H.G. & Guerra, R.O., 2012. The role of maternal socioeconomic factors in the commitment to universal newborn hearing screening in the Northeastern region of Brazil. *International Journal Of Pediatric Otorhinolaryngology*, 76(11), pp.1661–1667. Available at: http://search.ebscohost.com/login.aspx?direct=true&db=cmedm&AN=22921603&site=ehost-live. |
| 278 | Cavalli, A. et al., 2010. Interactions between Global Health Initiatives and country health systems: the case of a neglected tropical diseases control program in Mali. *Plos Neglected Tropical Diseases*, 4(8), pp.e798–e798. Available at: http://search.ebscohost.com/login.aspx?direct=true&db=cmedm&AN=20808908&site=ehost-live. |
| 279 | Charlés, L.L., 2015. Scaling Up Family Therapy in Fragile, Conflict-Affected States. *Family Process*, 54(3), pp.545–558. Available at: http://search.ebscohost.com/login.aspx?direct=true&db=cmedm&AN=25315510&site=ehost-live. |
| 280 | Chaturvedi, S. et al., 2016. Time-constrained mother and expanding market: emerging model of under-nutrition in India. *BMC Public Health*, 16, p.632. Available at: http://search.ebscohost.com/login.aspx?direct=true&db=cmedm&AN=27456223&site=ehost-live. |
| 281 | Chetty, V. & Hanass-Hancock, J., 2016. A rehabilitation model as key to comprehensive care in the era of HIV as a chronic disease in South Africa. *AIDS Care*, 28 Suppl 1, pp.132–139. Available at: http://search.ebscohost.com/login.aspx?direct=true&db=cmedm&AN=27002771&site=ehost-live. |
| 282 | Chima, C.C. & Homedes, N., 2015. Impact of global health governance on country health systems: the case of HIV initiatives in Nigeria. *Journal Of Global Health*, 5(1), p.10407. Available at: http://search.ebscohost.com/login.aspx?direct=true&db=cmedm&AN=25969731&site=ehost-live. |
| 283 | Clancy, T.R., 2015. Complexity, flow, and antifragile healthcare systems: implications for nurse executives. *The Journal Of Nursing Administration*, 45(4), pp.188–191. Available at: http://search.ebscohost.com/login.aspx?direct=true&db=cmedm&AN=25803798&site=ehost-live. |
| 284 | Clarke, J.N. & Fletcher, P.C., 2004. Parents as advocates: stories of surplus suffering when a child is diagnosed and treated for cancer. *Social Work In Health Care*, 39(1–2), pp.107–127. Available at: http://search.ebscohost.com/login.aspx?direct=true&db=cmedm&AN=15774387&site=ehost-live. |
| 285 | Brennan-Ing, M. et al., 2014. Service utilization among older adults with HIV: the joint association of sexual identity and gender. *Journal Of Homosexuality*, 61(1), pp.166–196. Available at: http://search.ebscohost.com/login.aspx?direct=true&db=cmedm&AN=24313258&site=ehost-live. |
| 286 | Brito-Silva, K. et al., 2014. Integrality in cervical cancer care: evaluation of access. *Revista De Saude Publica*, 48(2), pp.240–248. Available at: http://search.ebscohost.com/login.aspx?direct=true&db=cmedm&AN=24897045&site=ehost-live. |
| 287 | Brooks, A. et al., 2012. Aligning new interventions with developing country health systems: target product profiles, presentation, and clinical trial design. *Global Public Health*, 7(9), pp.931–945. Available at: http://search.ebscohost.com/login.aspx?direct=true&db=cmedm&AN=22783872&site=ehost-live. |
| 288 | Burkle Jr, F.M., 2017. The Politics of Global Public Health in Fragile States and Ungoverned Territories. *Plos Currents*, 9. Available at: http://search.ebscohost.com/login.aspx?direct=true&db=cmedm&AN=28228975&site=ehost-live. |
| 289 | Buse, K. & Walt, G., 1996. Aid coordination for health sector reform: a conceptual framework for analysis and assessment. *Health Policy (Amsterdam, Netherlands)*, 38(3), pp.173–187. Available at: http://search.ebscohost.com/login.aspx?direct=true&db=cmedm&AN=10162420&site=ehost-live. |
| 290 | Byrne, A., Hodge, A. & Jimenez-Soto, E., 2015. Accelerating Maternal and Child Health Gains in Papua New Guinea: Modelled Predictions from Closing the Equity Gap Using LiST. *Maternal And Child Health Journal*, 19(11), pp.2429–2437. Available at: http://search.ebscohost.com/login.aspx?direct=true&db=cmedm&AN=26108400&site=ehost-live. |
| 291 | Calain, P., 2007. From the field side of the binoculars: a different view on global public health surveillance. *Health Policy And Planning*, 22(1), pp.13–20. Available at: http://search.ebscohost.com/login.aspx?direct=true&db=cmedm&AN=17237490&site=ehost-live. |
| 292 | Caldwell, J.C., Orubuloye, I.O. & Caldwell, P., 1992. Underreaction to AIDS in Sub-Saharan Africa. *Social Science & Medicine (1982)*, 34(11), pp.1169–1182. Available at: http://search.ebscohost.com/login.aspx?direct=true&db=cmedm&AN=1641678&site=ehost-live. |
| 293 | Casarett, D.J. & Lantos, J.D., 1998. Have we treated AIDS too well? Rationing and the future of AIDS exceptionalism. *Annals Of Internal Medicine*, 128(9), pp.756–759. Available at: http://search.ebscohost.com/login.aspx?direct=true&db=cmedm&AN=9556470&site=ehost-live. |
| 294 | Cleaveland, S. et al., 2017. One Health contributions towards more effective and equitable approaches to health in low- and middle-income countries. *Philosophical Transactions Of The Royal Society Of London. Series B, Biological Sciences*, 372(1725). Available at: http://search.ebscohost.com/login.aspx?direct=true&db=cmedm&AN=28584176&site=ehost-live. |
| 295 | Cliquet, F., Picard-Meyer, E. & Robardet, E., 2014. Rabies in Europe: what are the risks? *Expert Review Of Anti-Infective Therapy*, 12(8), pp.905–908. Available at: http://search.ebscohost.com/login.aspx?direct=true&db=cmedm&AN=24847903&site=ehost-live. |
| 296 | Cohen, J.M. et al., 2012. Malaria resurgence: a systematic review and assessment of its causes. *Malaria Journal*, 11, p.122. Available at: http://search.ebscohost.com/login.aspx?direct=true&db=cmedm&AN=22531245&site=ehost-live. |
| 297 | Craig, B.J. & Kapysheva, A., 2017. Situated influences on perceived barriers to health behavior change: cultural identity and context in Kazakhstan. *Ethnicity & Health*, pp.1–16. Available at: http://search.ebscohost.com/login.aspx?direct=true&db=cmedm&AN=28277025&site=ehost-live. |
| 298 | Croll, P.R. & Croll, J., 2007. Investigating risk exposure in e-health systems. *International Journal Of Medical Informatics*, 76(5–6), pp.460–465. Available at: http://search.ebscohost.com/login.aspx?direct=true&db=cmedm&AN=17126069&site=ehost-live. |
| 299 | Baird, M. (2011). *Service Delivery in Fragile and Conflict-Affected States*. Washington, DC: World Bank. Available From: https://openknowledge.worldbank.org/handle/10986/9203 [Accessed 7th November 2018) |
| 300 | Cali, M. (2014). *A Fragile Country Tale: Restrictions, Trade Deficits, and Aid Dependence*. Available from: http://blogs.worldbank.org/trade/fragile-country-tale-restrictions-trade-deficits-and-aid-dependence [Accessed 8th November 2018] |
| 301 | Marc, A. et al. (2013). *Societal Dynamics and Fragility: Engaging societies in responding to fragile situations.* New frontiers of social policy. Washington, DC: World Bank. Available from: https://openknowledge.worldbank.org/handle/10986/12222 [Accessed 8th November 2018] |
| 302 | Gates, S. et al. (2010). *Consequences of Civil Conflict*. Washington, DC: World Bank. Available from: https://openknowledge.worldbank.org/handle/10986/9071 [Accessed 8th November 2018] |
| 303 | Tartir, A. (2015). *Can fragility in countries be addressed outside of politics?* Available from: http://blogs.worldbank.org/arabvoices/can-fragility-countries-be-addressed-outside-politics [Accessed 8th November 2018] |
| 304 | Indrawati, S.M. (2016). *How we're fighting conflict and fragility where poverty is deepest*. Available from: https://blogs.worldbank.org/voices/how-we-re-fighting-conflict-and-fragility-where-poverty-deepest [Accessed 8th November 2018] |
| 305 | Philips, M. & Derderian, K. (2015). Health in the service of state-building in fragile and conflict affected contexts: an additional challenge in the medical-humanitarian environment. *Conflict and Health*. 9:13. Available from: https://doi.org/10.1186/s13031-015-0039-4 |
| 306 | Ager, A. et al. (2014). Health service resilience in Yobe state, Nigeria in the context of the Boko Haram insurgency: a systems dynamics analysis using group model building. *Conflict and Health.* 9:30. Available from: https://doi.org/10.1186/s13031-015-0056-3 |
| 307 | Percival, V. et al. (2015). Health systems and gender in post-conflict contexts: building back better? *Conflict and Health*. 8:19. Available from: https://doi.org/10.1186/1752-1505-8-19 |
| 308 | Jerpseth, H. et al., 2017. Older patients with late-stage COPD: their illness experiences and involvement in decision-making regarding mechanical ventilation and noninvasive ventilation. *Journal Of Clinical Nursing*. Available at: http://search.ebscohost.com/login.aspx?direct=true&db=cmedm&AN=28618112&site=ehost-live. |
| 309 | Kehr, J., 2017. “Exotic no more”: Tuberculosis, public debt and global health in Berlin. *Global Public Health*, pp.1–14. Available at: http://search.ebscohost.com/login.aspx?direct=true&db=cmedm&AN=28786324&site=ehost-live. |
| 310 | Kizer, K.W., 2000. Lessons learned in public health emergency management: personal reflections. *Prehospital And Disaster Medicine*, 15(4), pp.209–214. Available at: http://search.ebscohost.com/login.aspx?direct=true&db=cmedm&AN=11227611&site=ehost-live. |
| 311 | Kottow, M.H., 2003. The vulnerable and the susceptible. *Bioethics*, 17(5–6), pp.460–471. Available at: http://search.ebscohost.com/login.aspx?direct=true&db=cmedm&AN=14959710&site=ehost-live. |
| 312 | Kulane, A. et al., 2016. Health in a fragile state: a five-year review of mortality patterns and trends at Somalia’s Banadir Hospital. *International Journal Of General Medicine*, 9, pp.303–310. Available at: http://search.ebscohost.com/login.aspx?direct=true&db=cmedm&AN=27621664&site=ehost-live. |
| 313 | Kumar, S. & Willman, A., 2016. Healing invisible wounds and rebuilding livelihoods: Emerging lessons for combining livelihood and psychosocial support in fragile and conflict-affected settings. *Journal Of Public Health Policy*, 37 Suppl 1, pp.32–50. Available at: http://search.ebscohost.com/login.aspx?direct=true&db=cmedm&AN=27638241&site=ehost-live. |
| 314 | Laverack, G. & Manoncourt, E., 2016. Key experiences of community engagement and social mobilization in the Ebola response. *Global Health Promotion*, 23(1), pp.79–82. Available at: http://search.ebscohost.com/login.aspx?direct=true&db=cmedm&AN=26518037&site=ehost-live. |
| 315 | Jacobstein, R. et al. (2009). Fragile, threatened, and still urgently needed: family planning programs in sub-Saharan Africa. *Studies in Family Planning*. 40(2): 147-154. Available from: https://doi.org/10.1111/j.1728-4465.2009.00197.x |
| 316 | Department for International Development. (2012). *Results in Fragile and Conflict-Affected States and Situations*. Available from: https://www.gov.uk/government/publications/results-in-fragile-and-conflict-affected-states-and-situations [Accessed 7th November 2018] |
| 317 | Department for International Development. (2010). *Briefing note: Working effectively in conflict-affected and fragile stituations: Paper A*. Available from: https://www.gov.uk/government/publications/briefing-note-working-effectively-in-conflict-affected-and-fragile-stituations-paper-a [Accessed 7th November 2018] |
| 318 | Department for International Development. (2010). *Synthesis of country programme evaluations conducted in fragile states (Ev709)*. Available from: https://www.gov.uk/government/publications/synthesis-of-country-programme-evaluations-conducted-in-fragile-states-ev709 [Accessed 7th November 2018] |
| 319 | Department for International Development. (2010). *DFID How to note on corruption in health*. Available from: https://www.gov.uk/government/publications/dfid-how-to-note-on-corruption-in-health [Accessed 7th November 2018] |
| 320 | Department for International Development. (2013). *Evaluation of Sierra Leone’s Youth Reproductive Health Programme 2007–2012*. Available from: https://www.gov.uk/government/publications/evaluation-of-sierra-leones-youth-reproductive-health-programme-2007-2012 [Accessed 7th November 2018] |
| 321 | Department for International Development. (2015). *DFID Annual Report and Accounts 2014-15 Results achieved by sector: Health*. Available from: https://www.gov.uk/government/publications/dfid-annual-report-and-accounts-2014-15-results-achieved-by-sector-health [Accessed 7th November 2018] |
| 322 | Ovadiya, M. (2015). *Social protection in fragile and conflict-affected countries : trends and challenges*. Social protection and labor discussion paper; no. 1502. Washington, D.C.: World Bank Group. Available from: http://documents.worldbank.org/curated/en/951221468185039094/Social-protection-in-fragile-and-conflict-affected-countries-trends-and-challenges [Accessed 7th November 2018] |
| 323 | International Development Association. (2007). *Operational Approaches and Financing in Fragile States.* Available from: http://siteresources.worldbank.org/IDA/Resources/IDA15FragileStates.pdf [Accessed 7th November 2018] |
| 324 | Demirgüç-Kunt, A. et al. (2013). *Financial Inclusion in Fragile and Conflict-Affected States*. Available from: http://blogs.worldbank.org/allaboutfinance/financial-inclusion-in-fragile-and-conflict-affected-states [Accessed 7th November 2018] |
| 325 | Atanesyan, K. (2017). *How to Tackle Global Fragility and Violence - Focus on Middle-Income Countries.* Available from: https://ieg.worldbankgroup.org/blog/how-tackle-global-fragility-and-violence-focus-middle-income-countries [Accessed 7th November 2018] |
| 326 | Goodhand, J. & Sedra, M., 2010. Who owns the peace? Aid, reconstruction, and peacebuilding in Afghanistan. *Disasters*, 34 Suppl 1, pp.S78–S102. Available at: http://search.ebscohost.com/login.aspx?direct=true&db=cmedm&AN=19486353&site=ehost-live. |
| 327 | Gordon, S., 2011. Health, stabilization and securitization: towards understanding the drivers of the military role in health interventions. *Medicine, Conflict, And Survival*, 27(1), pp.43–66. Available at: http://search.ebscohost.com/login.aspx?direct=true&db=cmedm&AN=21721347&site=ehost-live. |
| 328 | Gruber, J., 2009. Technical assistance for health in non-conflict fragile states: challenges and opportunities. *The International Journal Of Health Planning And Management*, 24 Suppl 1, pp.S4–S20. Available at: http://search.ebscohost.com/login.aspx?direct=true&db=cmedm&AN=19957309&site=ehost-live. |
| 329 | Gursky, E.A., 2015. Rising to the Challenge: The Ebola Outbreak in Sierra Leone and How Insights Into One Nongovernmental Organization’s Response Can Inform Future Core Competencies. *Disaster Medicine And Public Health Preparedness*, 9(5), pp.554–557. Available at: http://search.ebscohost.com/login.aspx?direct=true&db=cmedm&AN=26330281&site=ehost-live. |
| 330 | Fotaki, M., 2011. Towards developing new partnerships in public services: users as consumers, citizens and/or co-producers in health and social care in England and Sweden. *Public Administration*, 89(3), pp.933–955. Available at: http://search.ebscohost.com/login.aspx?direct=true&db=cmedm&AN=22165151&site=ehost-live. |
| 331 | Harrikari, T., 2014. Social disorganization and the profile of child welfare: Explaining child welfare activity by the community-level factors. *Child Abuse & Neglect*, 38(10), pp.1671–1682. Available at: http://search.ebscohost.com/login.aspx?direct=true&db=cmedm&AN=25082431&site=ehost-live. |
| 332 | Haugh, R., 2002. By a thread--a fragile, fraying safety net is everybody’s problem. *Hospitals & Health Networks*, 76(6), p.34. Available at: http://search.ebscohost.com/login.aspx?direct=true&db=cmedm&AN=12080920&site=ehost-live. |
| 333 | Holmes, M., 2015. Financially fragile rural hospitals: mergers and closures. *North Carolina Medical Journal*, 76(1), pp.37–40. Available at: http://search.ebscohost.com/login.aspx?direct=true&db=cmedm&AN=25621479&site=ehost-live. |
| 334 | Witter, S. (2012). Health financing in fragile and post-conflict states: What do we know and what are the gaps?. *Social Science and Medicine*. 75(12): 2370-2377. Available from: https://doi.org/10.1016/j.socscimed.2012.09.012 |
| 335 | Bhatnagar, A. & George, A.S., 2016. Motivating health workers up to a limit: partial effects of performance-based financing on working environments in Nigeria. *Health Policy and Planning*, 31(7), pp.868–877. Available at: https://doi.org/10.1093/heapol/czw002 |
| 336 | Dalmar, A.A. et al., 2017. Rebuilding research capacity in fragile states: the case of a Somali-Swedish global health initiative. *Global Health Action*, 10(1), p.1348693. Available at: http://search.ebscohost.com/login.aspx?direct=true&db=cmedm&AN=28799463&site=ehost-live. |
| 337 | de Negri Filho, A., 2008. A human rights approach to quality of life and health: applications to public health programming. *Health And Human Rights*, 10(1), pp.93–101. Available at: http://search.ebscohost.com/login.aspx?direct=true&db=cmedm&AN=20845832&site=ehost-live. |
| 338 | Delph, Y.M., 1993. Health priorities in developing countries. *The Journal Of Law, Medicine & Ethics: A Journal Of The American Society Of Law, Medicine & Ethics*, 21(1), pp.16–22. Available at: http://search.ebscohost.com/login.aspx?direct=true&db=cmedm&AN=11652118&site=ehost-live. |
| 339 | Dodd, R. & Lane, C., 2010. Improving the long-term sustainability of health aid: are Global Health Partnerships leading the way? *Health Policy and Planning*, 25(5), pp. 363–371. Available at: https://doi.org/10.1093/heapol/czq014 |
| 340 | Du Mortier, S. et al., 2016. A decade of an HIV workplace programme in armed conflict zones; a social responsibility response of the International Committee of the Red Cross. *Journal Of Occupational Medicine And Toxicology (London, England)*, 11, p.28. Available at: http://search.ebscohost.com/login.aspx?direct=true&db=cmedm&AN=27247611&site=ehost-live. |
| 341 | Dynes, M.M. et al., 2015. Perceptions of the risk for Ebola and health facility use among health workers and pregnant and lactating women--Kenema District, Sierra Leone, September 2014. *MMWR. Morbidity And Mortality Weekly Report*, 63(51), pp.1226–1227. Available at: http://search.ebscohost.com/login.aspx?direct=true&db=cmedm&AN=25551595&site=ehost-live. |
| 342 | Erwin, D.P. et al., 2011. Challenges and implementation of a women’s breast health initiative in rural Kashmir. *Breast (Edinburgh, Scotland)*, 20 Suppl 2, pp.S46–S50. Available at: http://search.ebscohost.com/login.aspx?direct=true&db=cmedm&AN=21324696&site=ehost-live. |
| 343 | McMahon, S. A. et al., 2016. Healthcare providers on the frontlines: a qualitative investigation of the social and emotional impact of delivering health services during Sierra Leone’s Ebola epidemic. *Health Policy and Planning*, 31(9), pp.1232–1239. Available at: https://doi.org/10.1093/heapol/czw055 |
| 344 | Qirbi, N. & Ismail, S.A., 2017. Health system functionality in a low-income country in the midst of conflict: the case of Yemen, *Health Policy and Planning*, 32(6), pp.911–922. Available at: https://doi.org/10.1093/heapol/czx031 |
| 345 | Takian, A., et al., 2015. The experience of purchaser–provider split in the implementation of family physician and rural health insurance in Iran: an institutional approach, *Health Policy and Planning*, 30(10), pp.1261–1271. Available at: https://doi.org/10.1093/heapol/czu135 |
| 346 | da Costa, M.C., Lopes, M.J.M. & Soares, J. dos S.F., 2015. Public health agendas addressing violence against rural women - an analysis of local level health services in the State of Rio Grande do Sul, Brazil TT - Agendas públicas de saúde no enfrentamento da violência contra mulheres rurais - análise do nível loc. *Ciênc. saúde coletiva*, 20(5), pp.1379–1387. Available at: http://dx.doi.org/10.1590/1413-81232015205.04412014 |
| 347 | da Cunha, K.S. et al., 2016. Myocardial revascularization: factors intervening in the reference and counter-reference in Primary Health Care. *Revista Da Escola De Enfermagem Da U S P*, 50(6), pp.965–972. Available at: http://search.ebscohost.com/login.aspx?direct=true&db=cmedm&AN=28198962&site=ehost-live. |
| 348 | Dalil, S. et al., 2014. Aid effectiveness in rebuilding the Afghan health system: a reflection. *Global Public Health*, 9 Suppl 1, pp.S124–S136. Available at: http://search.ebscohost.com/login.aspx?direct=true&db=cmedm&AN=24922192&site=ehost-live. |
| 349 | Daoud, A., Halleröd, B. & Guha-Sapir, D., 2016. What Is the Association between Absolute Child Poverty, Poor Governance, and Natural Disasters? A Global Comparison of Some of the Realities of Climate Change. *Plos One*, 11(4), pp.e0153296–e0153296. Available at: http://search.ebscohost.com/login.aspx?direct=true&db=cmedm&AN=27077913&site=ehost-live. |
| 350 | David, P.-M., 2017. Measurement, “scriptural economies,” and social justice: governing HIV/AIDS treatments by numbers in a fragile state, the Central African Republic (CAR). *Developing World Bioethics*, 17(1), pp.32–39. Available at: https://doi.org/10.1111/dewb.12107 |
| 351 | de Castro, C.P. et al, 2016. Matrix Support in the SUS of Campinas: how an inter-professional practice has developed and consolidated in the health networkIntroduction. *Ciência & Saúde Coletiva*, 21(5), pp.1625-1636. Available at: https://dx.doi.org/10.1590/1413-81232015215.19302015 |
| 352 | de Souza, K.M.J. et al., 2010. [Tuberculosis treatment drop out and relations of bonding to the family health team]. *Revista Da Escola De Enfermagem Da U S P*, 44(4), pp.904–911. Available at: http://search.ebscohost.com/login.aspx?direct=true&db=cmedm&AN=21337770&site=ehost-live. |
| 353 | de Waal, A., 2010. Reframing governance, security and conflict in the light of HIV/AIDS: a synthesis of findings from the AIDS, Security and Conflict Initiative. *Social Science & Medicine (1982)*, 70(1), pp.114–120. Available at: http://search.ebscohost.com/login.aspx?direct=true&db=cmedm&AN=19819057&site=ehost-live. |
| 354 | del Valle, H. & Healy, S., 2013. Humanitarian agencies and authoritarian states: a symbiotic relationship? *Disasters*, 37 Suppl 2, pp.S188–S201. Available at: http://search.ebscohost.com/login.aspx?direct=true&db=cmedm&AN=23876075&site=ehost-live. |
| 355 | Derderian, K., 2014. Changing tracks as situations change: humanitarian and health response along the Liberia-Côte d’Ivoire border. *Disasters*, 38(4), pp.673–689. Available at: http://search.ebscohost.com/login.aspx?direct=true&db=cmedm&AN=25196331&site=ehost-live. |
| 356 | Desilva, M.B., Manworren, J. & Targonski, P., 2011. Impact of a housing first program on health utilization outcomes among chronically homeless persons. *Journal Of Primary Care & Community Health*, 2(1), pp.16–20. Available at: http://search.ebscohost.com/login.aspx?direct=true&db=cmedm&AN=23804657&site=ehost-live. |
| 357 | Dhakal, R., Ratanawijitrasin, S. & Srithamrongsawat, S., 2009. Addressing the challenges to health sector decentralization in Nepal: an inquiry into the policy and implementation processes. *Nepal Medical College Journal: NMCJ*, 11(3), pp.152–157. Available at: http://search.ebscohost.com/login.aspx?direct=true&db=cmedm&AN=20334060&site=ehost-live. |
| 358 | Doherty, T. et al., 2017. “If donors woke up tomorrow and said we can’t fund you, what would we do?” A health system dynamics analysis of implementation of PMTCT option B+ in Uganda. *Globalization And Health*, 13(1), p.51. Available at: http://search.ebscohost.com/login.aspx?direct=true&db=cmedm&AN=28747196&site=ehost-live. |
| 359 | Dos Reis, M.J., Lopes, M.H.B. de M. & Osis, M.J.D., 2017. “It's much worse than dying’: the experiences of female victims of sexual violence. *Journal Of Clinical Nursing*, 26(15–16), pp.2353–2361. Available at: https://doi.org/10.1111/jocn.13247 |
| 360 | Drobac, P. & Morse, M., 2016. Medical Education and Global Health Equity. *AMA Journal Of Ethics*, 18(7), pp.702–709. Available at: http://search.ebscohost.com/login.aspx?direct=true&db=cmedm&AN=27437820&site=ehost-live. |
| 361 | Durham, J. et al., 2015. Haïti and the health marketplace: the role of the private, informal market in filling the gaps left by the state. *BMC Health Services Research*, 15, p.424. Available at: http://search.ebscohost.com/login.aspx?direct=true&db=cmedm&AN=26416252&site=ehost-live. |
| 362 | Duvivier, R.J., Burch, V.C. & Boulet, J.R., 2017. A comparison of physician emigration from Africa to the United States of America between 2005 and 2015. *Human Resources For Health*, 15(1), p.41. Available at: http://search.ebscohost.com/login.aspx?direct=true&db=cmedm&AN=28651539&site=ehost-live. |
| 363 | Dwyer, R. & Fraser, S., 2017. Engendering drug problems: Materialising gender in the DUDIT and other screening and diagnostic “apparatuses”. *The International Journal On Drug Policy*, 44, pp.135–144. Available at: http://search.ebscohost.com/login.aspx?direct=true&db=cmedm&AN=28641975&site=ehost-live. |
| 364 | Edward, A. et al., 2015. Enhancing governance and health system accountability for people centered healthcare: an exploratory study of community scorecards in Afghanistan. *BMC Health Services Research*, 15, p.299. Available at: http://search.ebscohost.com/login.aspx?direct=true&db=cmedm&AN=26227814&site=ehost-live. |
| 365 | Egami, Y. et al., 2012. Can health systems be enhanced for optimal health services through disease-specific programs? Results of field studies in Viet Nam and Cambodia. *Bioscience Trends*, 6(1), pp.1–6. Available at: http://search.ebscohost.com/login.aspx?direct=true&db=cmedm&AN=22426096&site=ehost-live. |
| 366 | Enlow, E. et al., 2017. Perspectives of Low Socioeconomic Status Mothers of Premature Infants. *Pediatrics*, 139(3). Available at: http://search.ebscohost.com/login.aspx?direct=true&db=cmedm&AN=28223372&site=ehost-live. |
| 367 | Far, F. El, Marino, C.G.J. & Medeiros, E.A.S., 2001. The organization of hospital infection control committees and their importance in Brazil. *Braz J Infect Dis*, 5(6), pp.290–293. Available at: http://www.scielo.br/scielo.php?script=sci_arttext&pid=S1413-86702001000600001&lng=pt&nrm=iso&tlng=en. |
| 368 | Fox, S. et al., 2014. Paying health workers for performance in a fragmented, fragile state: reflections from Katanga Province, Democratic Republic of Congo, *Health Policy and Planning*, 29(1), pp.96–105. Available at: https://doi.org/10.1093/heapol/czs138 |
| 369 | Lee, K. & Brumme, Z.L., 2013. Operationalizing the One Health approach: the global governance challenges. *Health Policy and Planning*, 28(7), pp.778–785. Available at: https://doi.org/10.1093/heapol/czs127 |
| 370 | Levin, A. & Kaddar, M., 2011. Role of the private sector in the provision of immunization services in low- and middle-income countries. *Health Policy and Planning*, 26(suppl 1), pp.i4–i12. Available at: https://doi.org/10.1093/heapol/czr037 |
| 371 | Meessen, B. et al., 2011. Removing user fees in the health sector: a review of policy processes in six sub-Saharan African countries. *Health Policy and Planning*, 26(suppl 2), pp.ii16–ii29. Available at: https://doi.org/10.1093/heapol/czr062 |
| 372 | Sarang, A. et al., 2013. Systemic barriers accessing HIV treatment among people who inject drugs in Russia: a qualitative study. *Health Policy and Planning*, 28(7), pp.681–691. Available at: https://doi.org/10.1093/heapol/czs107 |
| 373 | Sax, S. & Marx, M., 2014. Local perceptions on factors influencing the introduction of international healthcare accreditation in Pakistan. *Health Policy and Planning*, 29(8), pp.1021–1030. Available at: https://doi.org/10.1093/heapol/czt084 |
| 374 | Silva, R.M.M.d. et al., 2015. Challenges in the coordination of children's healthcare. *Ciência & Saúde Coletiva*, 20(4), pp.1217-1224. Available at: https://dx.doi.org/10.1590/1413-81232015204.00742014 |
| 375 | Tkatchenko-Schmidt, E. et al., 2010. Why do health systems matter? Exploring links between health systems and HIV response: a case study from Russia. *Health Policy and Planning*, 25(4), pp.283–291. Available at: https://doi.org/10.1093/heapol/czq001 |
| 376 | Witter, S. et al., 2017. Minding the gaps: health financing, universal health coverage and gender. *Health Policy and Planning*, 32(suppl 5), pp.v4–v12. Available at: https://doi.org/10.1093/heapol/czx063 |
| 377 | Witter, S. et al., 2016. The free health care initiative: how has it affected health workers in Sierra Leone? *Health Policy and Planning*, 31(1), pp.1–9. Available at: https://doi.org/10.1093/heapol/czv006 |
