# Supplementary material for: Understanding fragility: implications for global health research and practice
Source: Health Policy Plan. 2019 Dec 10;35(2):235–43. doi: 10.1093/heapol/czz142 (PMC7050687; doi:10.1093/heapol/czz142)
Supplement: czz142_Supplementary_Data [file czz142_supplementary_data.zip › czz142-suppl_data/02_Supplementary File 4_Narrative synthesis.docx]

**Understanding fragility: implications for global health research and practice: Narrative Synthesis**

| Settings noted as 'fragile' (no definition provided, n=44) and/or as experiencing severe chronic challenges resulting in repeated outbreaks of violence (n=4) | | | |
| --- | --- | --- | --- |
| **Overview of settings included in this category of studies** | | | Settings are historically experiencing frequent bouts of war, conflict and violence and resulting substantive displacement; society is fragmented, physical infrastructure compromised at scale (e.g. as in 43, 46, 62, 77, 80, 89, 103, 137, 188, 255, 312, 377). Contexts experience long-lasting political conflict that result in dramatic shifts in economic conditions at population level (e.g. as in e.g. shifting safety nets - 120) (e.g. as in 119, 114) and are further potentially affected by environmental disasters (e.g. as in 310). |
| **Narrative synthesis of fragility applications by referent** | **Health system** | **Finance** | Limited resources (e.g. as in 71, 152, 376); high out of pocket expenditures (e.g. as in 71); lack of system transparency and potential misuse of funds (e.g. as in 71, 80); limited preparedness for crisis - no budgets (e.g. as in 71); limited long-term financial commitments from donors, other agencies (e.g. as in 79, 80, 120, 273) |
|  |  | **Governance** | Limited policies and direction relating to human resource capacity building, management, remuneration (e.g. as in 53, 71, 56); lack of national policies and resulting clinical guidelines (e.g. as in 53, 124); limited management capacity in the system (e.g. as in 71, 77, 80); mismatch in donor priorities and national priorities potentially leading to initiatives tailored to 'perceived need' (e.g. as in 89, 144); need to strengthen inter-sectoral governance given spillovers of conflict (e.g. as in 89, 188, 273); general emphasis on harmonization among actors (e.g. as in donors, national, service delivery) in promoting stability and long-term engagement beyond humanitarian response (e.g. as in 119, 120, 144, 326, 327) |
|  |  | **Information systems** | Poor information systems, including surveillance, and logistics (e.g. as in 53, 80, 94, 210) |
|  |  | **Human resources** | (e.g. as in Note also effects of conflict on communities) Low number of health care workers (e.g. as in 46, 53, 71, 77, 80, 89); limited skills among health care workers (e.g. as in 62); limited remuneration and potentially dysfunctional incentives (e.g. as in 53) |
|  |  | **Service delivery** | Limited capacity of national systems to deliver services, resulting in contracting (e.g. as in 121, 255) or international intervention (e.g. as in 326); inappropriate clinical practice due to lack of knowledge and/or coping mechanisms becoming entrenched (e.g. as in 124, 46); general difficulty in delivery of services due to infrastructure (e.g. as in 46, 178); service offer skewed towards specific priorities - e.g. RMCH (e.g. as in 200) |
|  |  | **Infrastructure** | Limited sanitation, medication and equipment (e.g. as in 53, 62, 80) |
|  | **Health system and community interface** | | Political processes shape both service delivery and utilization of services (e.g. as in 43); health service delivery and provider practices are grounded in cultural norms and may be discriminatory and iniquitous (e.g. as in 62, 273); limited trust in health system and wider political systems (e.g. as in 94); emphasis on securing local engagement and empowering communities in order to deliver care and build local credibility (e.g. as in 79, 80, 87) |
|  | **Community system** | | Populations living in extreme poverty and/or being forced into migration due to economic/conflict conditions (e.g. as in 46, 89, 210); difficulties in accessing care in general (e.g. as in 87,250, 299) but specifically due to poor road conditions, inability to secure transport (e.g. as in 53, 62, 210) and/or lack of resources/fears of incurring expenditure (e.g. as in 62, 137, 152) and/or general lack of information, cultural beliefs and fear of health care worker discrimination (e.g. as in 62); precarious living conditions lead to inability to control own financing capacity (e.g. as in earning, consumption 324, 326, 336) and result in coping practices that do not promote healthy living and wellbeing (e.g. as in 161); women and children are recognized as vulnerable populations (e.g. as in 82) |

| **Settings noted to be affected by time-bound episodes of conflict and violence which result in humanitarian crises (n=49)** | | | |
| --- | --- | --- | --- |
| **Overview of settings included in this category of studies** | | | Termed "challenging operating environments" these are settings where armed conflict and prolonged violence lead to erosion of physical infrastructure and substantive security challenges. Crime, interpersonal violence and ethnic conflicts are frequent. There is a risk of radicalization of the populace and democracy is frail. (e.g. as in 254) Violence and crime result in the death and/or migration and displacement of communities and health staff. (e.g. as in 1, 4, 6, 33, 34, 15, 16, 17, 26, 28, 207, 9, 270, 300, 306, 76, 325) |
| **Narrative synthesis of fragility applications by referent** | **Health system** | **Finance** | Costs of running services in insecure conditions is high (e.g. as in 5) as are user fees (e.g. as in 33). There is generally limited financing capacity (e.g. as in 34) and over-reliance on external aid (e.g. as in 97, 83); no transparency in the use of funds (e.g. as in 13) |
|  |  | **Governance** | National governance capacity (e.g. as in including in the health system) is limited (e.g. as in 74, 83, 269, 307). When present, governance capacity is fragmented and characterized by hierarchical leadership (e.g. as in 3) creating a governance system that is not accountable and potentially corrupt (e.g. as in 13). Local and informed leadership is lacking (e.g. as in 26); severe lack of exert knowledge and engagement (e.g. as in 19, 34). Policies are overly restrictive and bureaucratic (e.g. as in 4, 20, 10) - for example resulting in inadequate staff distribution (e.g. as in 7,19) and or impeding work by CHWs (e.g. as in 10). Intersectoral and donor engagement is difficult; donors are likely to pursue a humanitarian and stabilization agenda but coordination of local actors is difficult and further harmonization is needed to engage partners and frontline health workers in service planning. (e.g. as in 3, 4, 14, 28) |
|  |  | **Information systems** | Data on both population and disease profile as well as service delivery is severely lacking (e.g. as in 4, 17, 13, 6, 270) |
|  |  | **Human resources** | Conflict and violence result in health workers deaths and migration (e.g. as in 6,7,10, 17, 19, 33, 34, 73, 76, 83, 265, 270, 288); absence of HR attraction, retention, training an remuneration policy is frequent (e.g. as in 15) which contributes to lack of skilled staff (e.g. as in 4, 20, 33, 34) and prompts staff absenteeism (e.g. as in 13). |
|  |  | **Service delivery** | Limited integration of services (e.g. as in 4) potentially prompted by misinformation and confusion on policies that are applicable to health service delivery (e.g. as in 4) and over-reliance on delivery by humanitarian actors (e.g. as in 17, 74, 83); services are delivered during conflict if experience and knowledge exists from pre-conflict periods (e.g. as in 4); services prioritize specific locations (e.g. as in e.g. refugee camps 28) and/or groups (e.g. as in e.g. gender 5); focus is clinical rather than public health (e.g. as in 34) - both types of services, including referral pathways are needed. (e.g. as in 63) |
|  |  | **Infrastructure** | Infrastructure is destroyed and depleted (e.g. as in 6, 33, 4, 16, 61, 83, 73, 235, 270, 265) medication and consumable supply is disrupted (e.g. as in 17, 20, 83, 265, 288) |
|  | **Health system and community interface** | | Negative care experiences (e.g. as in e.g. due to poor health worker attitudes 4, 9 and fears of stigmatization and mis-treatment 9) lead to fears of accessing care and non-utilization of services (e.g. as in 4, 9). Culture related barriers to service access may exist (e.g. as in e.g. for women accessing services unaccompanied) and fears of the confidentiality maintained by care workers affect perceptions of care quality and trust. (e.g. as in 20, 325, 76) Dedicated grievance or complaint mechanisms are absent and structures for health system community dialogue are minimal (e.g. as in 13). Community health workers are viewed as one strategy to ensure community engagement (e.g. as in 10, 20), however they may then unfairly shoulder the blame for health system 'failings' (e.g. as in 10). General low trust in service provision and formal systems (e.g. as in 13) and limited acknowledgement of long-term emotional societal hurt (e.g. as in 21). |
|  | **Community system** | | Community systems are fragmented due to violence: there are difficulties in integrating perpetrators of violence (e.g. as in 5), resulting in social fragmentation (e.g. as in 34). There is a potential need for international security forces to aid in stabilization (e.g. as in 1). Poor living conditions (e.g. as in 34), particularly in rural areas, and social fragmentation prompt migration to urban more prosperous environments (e.g. as in 7). Access to care is difficult due to lack of education (e.g. as in girls specifically 7, 5, 22) and stresses of material poverty and potential additional expenses (e.g. as in 9). There is limited information available on service entitlements (e.g. as in 13) and geographic and climatic conditions make transport to/from health facilities difficult (e.g. as in 33). Women are viewed as a particularly vulnerable group (e.g. as in 22, 5) as are the urban poor, slum population and young. (e.g. as in 73) Violence related injuries, including gender based violence, are seen more frequently (e.g. as in 254) and result in feelings of helplessness in the population (e.g. as in 359) |

| **Settings exposed to diverse chronic political, economic and environmental challenges (n=51)** | | | |
| --- | --- | --- | --- |
| **Overview of settings included in this category of studies** | | | Settings are characterized by a generally weak and limited economic base, resulting in substantive poverty. (e.g. as in 48) Politically and socially, inequality is high - e.g. settings referenced often host refugee populations who are given limited rights (e.g. as in e.g. 29). Settings are environmentally fragile: poor conditions for agricultural development (e.g. as in 160), frequent natural disasters (e.g. as in 42) and environmental risks for zoonotic transmission is high. |
| **Narrative synthesis of fragility applications by referent** | **Health system** | **Finance** | Limited funding availability within the health system, and need for overseas development assistance (e.g. as in 68, 148); often vague resource allocation processes (e.g. as in 338) |
|  |  | **Governance** | Practical governance capacity is weak (e.g. as in 214, 282) and cross-sectoral linkages are needed to resolve existing issues (e.g. as in 294). There is limited organization of care (e.g. as in 68) and service delivery distortions may be introduced by donors via financing mechanisms (e.g. as in e.g. as relates to Global Fund and others prioritizing certain conditions 169, 282). In case of severe emergency, humanitarian actors may need to step in, however limited international governance structures exist to manage this in the case of 'non-fragile and conflict affected states' (e.g. as in 8). |
|  |  | **Information systems** | Limited to no information systems, particularly not for chronic care deliver (e.g. as in 68, 138) and information not trusted to be reliable. (e.g. as in 214) |
|  |  | **Human resources** | Over-reliance on community health worker programs, despite these workers dropping out at high rates due to community conditions (e.g. as in e.g. slum evictions) and health system inadequacy (e.g. as in e.g. no remuneration) (e.g. as in 35). Limited staff recruitment (e.g. as in and if done, aligned to vertical donor financed programs 169) and limited staff skills (e.g. as in 42, 214, 321). |
|  |  | **Service delivery** | Service delivery characterized by limited staff attention and long waiting times (e.g. as in 24) as well as competing delivery of vertical programs (e.g. as in 214, 282). If primary care established as only gateway to health system but proves ineffective, this is recognized as a problem (e.g. as in 68). Contracting out may be an option for service delivery if national capacity is limited (e.g. as in 24), particularly for delivery in rural areas. Models of care are inconsistent and may differ by location (e.g. as in e.g. public health in rural areas, curative in urban - 259). Outbreak management capacity is minimal given infrastructural challenges and may prompt international intervention (e.g. as in 314). |
|  |  | **Infrastructure** | No medicines or interrupted supply (e.g. as in 24, 68, 158, 214); limited health facility infrastructure (e.g. as in 24, 42, 321); limited or dysfunctional equipment (e.g. as in 68, 214, 321). If equipment available, likely due to parallel supply systems associated with donor funding (e.g. as in 282). |
|  | **Health system and community interface** | | Historical experience of public health services (e.g. as in 25) and personal experiences of poor quality care (e.g. as in 314) compromise community utilization of services. Trust in health systems is low (e.g. as in 294, 314) and rumours and conspiracy theories relating to why services are delivered often take hold in population (e.g. as in 25). Services and treatments are mis-matched to local cultural norms and circumstances - e.g. delivery of family planning to populations who culturally rely on large families (e.g. as in 261); limited responsiveness of systems when faced with these circumstances (e.g. as in 280, 276, 314). Health services are recognized as necessary for maintaining community life (e.g. as in e.g. for rural communities 246) and as prompting social change - e.g. family planning rights for women (e.g. as in 243) or advocacy for alleviating living conditions of poor (e.g. as in 276). There is emphasis on care networks (e.g. as in formal and informal providers and communities) for delivery of comprehensive care to vulnerable populations (e.g. as in 260, 281, 294); the elderly and HIV/TB populations are recognized as particularly vulnerable. (e.g. as in 110, 260, 281) |
|  | **Community system** | | Communities migrate frequently (e.g. as in 25, 261) due to economic and environmental conditions - e.g. populations live in crowded settlements such as slums, areas with limited opportunity to farm and/or strong seasonality in farming, areas naturally prone to natural disasters/or transmission of disease via insect and animal vectors. (e.g. as in 42, 60, 29, 86, 101, 160, 261, 280, 295, 343). Transmission of drug-resistant disease strains is highly likely (e.g. as in 296). In such locations, there is limited training available for the population, hence individuals' ability to secure their livelihood is compromised (e.g. as in 51, 60). Delays in care seeking are frequent (e.g. as in 280). Traditional health care access barriers present: financial access (e.g. as in 29, 48, 280, 281), geographic and transport related (e.g. as in 29, 158, 281), socio-political (e.g. as in recognition of rights and entitlements - 29, 141, 303), low levels of education (e.g. as in 51) and limited cultural recognition of what constitutes 'disease' (e.g. as in 25). Recognition of mothers and children presenting vulnerable populations. (e.g. as in 341) |

| **Settings not otherwise characterized as fragile (n=173)** | | | |
| --- | --- | --- | --- |
| **Overview of settings included in this category of studies** | | | Settings are not explicitly characterized as 'fragile', though populations in these settings my experience temporary stressors (e.g. as in e.g. poverty due to environmental shocks 57) |
| **Narrative synthesis of fragility applications by referent** | **Health system sphere** | **Finance** | Limited financial sustainability (e.g. as in 112, 251): countries and organizations rely heavily on donor support (e.g. as in 108) or themselves lack the resources to offer services sustainably (e.g. as in 230, 333) or enable adequate regulation (e.g. as in 257) . Corruption weakens financial systems and is notable as a challenge (e.g. as in 319). Limited financial planning and judicious resource allocation capacity: the multitude of donors may overwhelm and distort national systems (e.g. as in 223); coordinating donor activity and financing is rarely possible for national authorities. (e.g. as in 289, 291) Lack of judicious financial planning may incentivize perverse behaviours (e.g. as in e.g. harmful competition between health facilities - 122) |
|  |  | **Governance** | While health policies may exist, the link to implementation is clearly disrupted (e.g. as in 23). This may be due to policies being designed in a piece-meal fashion, ignoring larger system complexity (e.g. as in 138). Local leaders and technical experts are lacking and coordination challenges persist : technical expertise is often missing within national governments (e.g. as in 81) and there is a general lack of leadership on most issues. (e.g. as in 116, 117, 145, 230) Emergencies lead to a quick shift of priorities that may compromise existing structures (e.g. as in e.g. in the case of Ebola - 224). Coordination challenges are notable more generally (e.g. as in 122, 289). |
|  |  | **Information systems** | If data is available, this often corresponds to vertical programs (e.g. as in 23); coordination of data sources for surveillance purposes is problematic and in case of emergencies may lead to underestimation of epidemics. (e.g. as in 165). Routine data collection systems are absent or offering relatively poor data (e.g. as in 23, 244, 165); lack of data directly impedes the possibility of research and improvement of current systems. (e.g. as in 31, 40, 275) |
|  |  | **Human resources** | There is a severe lack of trained human resources: personnel is not available at facilities/in programs (e.g. as in 38, 132, 135), in limited supply generally in the labour market (e.g. as in 95, 154, 170) or when available subject to high turnover due to poor working conditions/contract types. (e.g. as in 95, 122, 362) Performance based financing programs may work to improve the situation if done alongside other infrastructural improvements and supervision. (335) |
|  |  | **Service delivery** | Inadequate planning of coordinated and equitous service delivery: there is poor geographic coverage of services (e.g. as in 18, 289, 290, 256), poor integration among programs and services (e.g. as in 49, 249, 278, 192, 347) including poor referral (e.g. as in 44, 249), poor consistency among similar services (e.g. as in 52, 176, 179). Services are inadequately managed (e.g. as in 230) and infrequently monitored. (e.g. as in 38). Notably, service gaps or lack of appropriate management are most visible in relation to infection, prevention and control (e.g. as in 147) and situations of overwhelming demand (e.g. as in 187). |
|  |  | **Infrastructure and equipment** | Limited availability of resources at both local facility levels as well as internationally (e.g. in the case of vaccines): laboratory infrastructure is limited and confined to certain geographic areas (e.g. as in 41), stockouts of medicines are frequent (e.g. as in 49) and commodities needed for service delivery are not available (e.g. as in 106, 150, 117, 168, 258, 264, 287, 335). |
|  | **Health system and community interface** | | Lack of trust in the health system and health care providers, as well as the care offered at health facilities, is a barrier to service access. (e.g. as in 67, 134, 147, 238, 263, 264) Patient and health service contact is frought with stressors: providers are often biased towards vulnerable groups/mistreat them (e.g. as in 18, 135, 157) and patients are acutely aware of the power imbalances between them and providers (e.g. as in e.g. families and patients often feel powerless as consent is not asked for before treatment initiation 125, 284). The tenseness of such situations leaves patients unwilling to discuss treatment options openly. (e.g. as in 44) |
|  | **Community sphere** | | Several vulnerable population groups stand out: Hispanic populations in the USA (e.g. as in 247), HIV patients (e.g. as in 54, 230, 241, 320, 163, 172, 171, 177, 205), end-of-life patients (e.g. as in 96, 135), the elderly and those of reduced mobility (e.g. as in 248, 285, 131, 133, 47, 50, 49), the chronically ill and those plagued by more than one illness. (e.g. as in 139, 104) Regional instability (e.g. as in 171, 173, 194, 239, 257, 196) and local limits to full social participation (e.g. as in 142, 175, 330) exacerbate the mistrust felt towards the health and any other public systems. |
|  |  |  | Vulnerable populations share a unique set of characteristics that act as factors for disease onset and stressors/barriers to improvement during periods of ill health. Low educational attainment is coupled with limited knowledge of healthy behaviours (e.g. as in 18, 32, 36, 44, 85) and poor lifestyles (e.g. as in 36, 150, 143, 155, 164, 183). Populations are trapped in poverty cycles with limited social capital to leverage - hence financial access barriers to health services are insurmountable (e.g. as in 236, 332, 309, 32, 49, 108, 131, 159, 192). Lack of education and limited family and social support (e.g. as in 164, 206, 47, 85, 131, 241, 133) reinforces a generic lack of hope and aspiration (e.g. as in 54, 163); at times this leads to crime (e.g. as in 241). To secure work, people migrate towards urban and industrial centres (e.g. as in 236); however both here and in rural areas housing conditions may be exceptionally poor. (e.g. as in 36, 131, 155, 164, 170, 277) Traveling to seek health care may be impossible due to travel conditions (e.g. as in 106, 159, 44, 49, 162) and additional religious (e.g. as in 18), culturo-linguistic (e.g. as in 18, 32, 107) and gender related barriers. (e.g. as in 157, 143, 225) |
